# Effect of Clinician Training in the Modular Approach to Therapy for Children vs Usual Care on Clinical Outcomes and Use of Empirically Supported Treatments: A Randomized Clinical Trial

**DOI:** 10.1001/jamanetworkopen.2020.11799

**Published:** 2020-08-17

**Authors:** Sally N. Merry, Sarah Hopkins, Mathijs F. G. Lucassen, Karolina Stasiak, John R. Weisz, Christopher M. A. Frampton, Sarah Kate Bearman, Ana M. Ugueto, Jennifer Herren, Ainsleigh Cribb-Su’a, Denise Kingi-Uluave, Jik Loy, Morgyn Hartdegen, Sue Crengle

**Affiliations:** 1 Department of Psychological Medicine, School of Medicine, FMHS University of Auckland, Auckland, New Zealand; 2 School of Health, Wellbeing and Social Care, The Open University, Milton Keynes, United Kingdom; 3 Department of Psychology, Harvard University, Cambridge, Massachusetts; 4 Department of Psychological Medicine, Christchurch School of Medicine, University of Otago, Christchurch, New Zealand; 5 Department of Educational Psychology, The University of Texas at Austin, Austin; 6 Department of Psychiatry and Behavioral Sciences, McGovern Medical School, The University of Texas Health Science Center at Houston, Houston; 7 Department of Psychiatry and Human Behavior, Alpert Medical School of Brown University, Providence, Rhode Island; 8 Le Va, Harakeke House, Manukau, Auckland, New Zealand; 9 Infant, Child and Adolescent Mental Health Services, Waikato District Health Board, Hamilton, New Zealand; 10 Department of Preventive and Social Medicine, Dunedin School of Medicine, University of Otago, Dunedin, New Zealand

## Abstract

**Importance:**

The Modular Approach to Therapy for Children (MATCH) was developed to address the comorbidities common among clinically referred youth, with beneficial outcomes shown in 2 US randomized clinical trials, where it outperformed both usual clinical care and single disorder–specific treatments.

**Objective:**

To determine whether MATCH training of clinicians would result in more use of empirically supported treatment (EST) and better clinical outcomes than usual care (UC) in the publicly funded, multidisciplinary context of New Zealand.

**Design, Setting, and Participants:**

This multisite, single-blind, computer-randomized clinical effectiveness trial compared MATCH with UC in child and adolescent mental health services in 5 regions of New Zealand. Recruitment occurred from March 2014 to July 2015, and a 3-month follow-up assessment was completed by May 2016. Clinicians at participating child and adolescent mental health services were randomized (1:1) to undertake training in MATCH or to deliver UC, and young people with anxiety, depression, trauma-related symptoms, or disruptive behavior seeking treatment at child and adolescent mental health services were randomized (1:1) to receive MATCH or UC. Participants and research assistants were blind to allocation. Data analysis was performed from April 2016 to July 2017.

**Interventions:**

MATCH comprises EST components for flexible management of common mental health problems. UC includes case management and psychological therapies. Both can include pharmacotherapy.

**Main Outcomes and Measures:**

There were 3 primary outcomes: trajectory of change of clinical severity, as measured by weekly ratings on the Brief Problem Monitor (BPM); fidelity to EST content, as measured by audio recordings of therapy sessions coded using the *Therapy Integrity in Evidence Based Interventions: Observational Coding System*; and efficiency of service delivery, as measured by duration of therapy (days) and clinician time (minutes).

**Results:**

The study included 65 clinicians (mean age, 38.7 years; range, 23.0-64.0 years; 54 female [83%]; MATCH, 32 clinicians; UC, 33 clinicians) and 206 young people (mean age, 11.2 years; range 7.0-14.0 years; 122 female [61%]; MATCH, 102 patients; UC, 104 patients). For the BPM total ratings for parents, there was a mean (SE) slope of –1.04 (0.14) (1-year change, −6.12) in the MATCH group vs –1.04 (0.10) (1-year change, −6.17) in the UC group (effect size, 0.00; 95% CI, −0.27 to 0.28; *P* = .96). For the BPM total for youths, the mean (SE) slope was –0.74 (0.15) (1-year change, −4.35) in the MATCH group vs –0.73 (0.10) (1-year change, −4.32) in the UC group (effect size, −0.02; 95% CI, −0.30 to 0.26; *P* = .97). Primary analyses (intention-to-treat) showed no difference in clinical outcomes or efficiency despite significantly higher fidelity to EST content in the MATCH group (58 coded sessions; mean [SD], 80.0% [20.0%]) than the UC group (51 coded sessions; mean [SD], 57.0% [32.0%]; *F*_(1,108)_ = 23.0; *P* < .001). With regard to efficiency of service delivery, there were no differences in total face-to-face clinician time between the MATCH group (mean [SD], 806 [527] minutes) and the UC group (mean [SD], 677 [539] minutes) or the overall duration of therapy between the MATCH group (mean [SD], 167 [107 days]) and the UC group (mean [SD], 159 [107] days).

**Conclusions and Relevance:**

MATCH significantly increased adherence to EST practices but did not improve outcomes or efficiency. The nonsuperiority of MATCH may be attributable to high levels of EST use in UC in New Zealand.

**Trial Registration:**

Australian New Zealand Clinical Trials Registry Identifier: ACTRN12614000297628

## Key Points

**Question:**

Is training in the Modular Approach to Therapy for Children (MATCH) associated with more use of empirically supported treatments, better clinical outcomes, and better service efficiency than usual care?

**Findings:**

This randomized clinical trial found that training clinicians in MATCH was associated with high levels of adherence to empirically supported treatments (80.0%) compared with usual care (57.0%), but it was not associated with improved clinical outcomes or efficiency.

**Meaning:**

These findings suggest that training in MATCH increases clinicians’ use of empirically supported treatments but does not necessarily improve clinical outcomes.

## Teaser

This randomized clinical trial evaluates whether clinician training in the Modular Approach to Therapy for Children (MATCH) is associated with more use of empirically supported treatment and better clinical outcomes than usual care among adolescents receiving mental health care in New Zealand.

## Introduction

Mental health problems in children and adolescents are common and persistent.^1,2^ There are effective therapies available^3^; however, delivering these therapies in clinical practice has been challenging.^4^ This is partly because the evidence is primarily available for single disorders or a homogeneous cluster of problems,^5^ whereas clinicians are faced with comorbid presentations that may change in focus during therapy. Clinicians may adopt a pragmatic but eclectic approach, unintentionally eroding the impact of carefully designed best clinical practice. The Modular Approach to Therapy for Children with Anxiety, Depression, Trauma, or Conduct Problems (MATCH-ADTC or MATCH, for brevity) has been designed to address the issues of flexibility and clinical complexity after a brief but comprehensive training program and has been shown to be more effective and efficient than usual care (UC).^6,7^

In New Zealand, preregistration courses for mental health professionals do not include in-depth training in psychological therapies for children and adolescents.^8^ For many working in child and adolescent mental health services (CAMHS), training in empirically supported treatments (ESTs), if it occurs, takes place after qualifying, either informally on the job or in courses that are time-consuming. Although there have been efforts to roll out training in ESTs in New Zealand, this is piecemeal so that having consistent delivery of ESTs is challenging.

We sought to determine whether training in MATCH could improve UC in New Zealand as it had been shown to do in studies in the US. Our primary hypotheses were that training CAMHS clinicians in MATCH, compared with UC, would increase the delivery of ESTs, improve clinical outcomes, and yield equal or better efficiency of service delivery.

## Methods

The study protocol has been published elsewhere^9^ and is available in Supplement 1. This study was approved by New Zealand’s Health and Disability Ethics Committee and was overseen by a Data Monitoring Committee of the Health Research Council of New Zealand. Participants provided written informed consent. This study follows the Consolidated Standards of Reporting Trials (CONSORT) reporting guideline.

### Trial Design

We performed a multisite, single-blind, randomized clinical effectiveness trial comparing MATCH with UC in CAMHS in 5 District Health Boards in New Zealand. In New Zealand, 20 District Health Boards are responsible for funding or providing health services within their district or geographical region. Participating teams in the District Health Boards provided services in rural and urban settings and included 1 Kaupapa Māori and 2 Pacific teams. Kaupapa Māori teams are those in which the philosophical doctrine incorporates the knowledge, skills, attitudes, and values of Māori (ie, Indigenous) society. Pacific teams are those in which people originating from other Pacific Islands are seen in services designed to incorporate the knowledge, skills, attitudes, and values of Pasifika societies.

Data were collected at baseline, during treatment, and 3 months after the end of treatment. The recruitment period was March 2014 to July 2015, with follow-up completed in May 2016.

### Randomization, Blinding, and Allocation Concealment

Randomization was at 2 levels: first, clinicians were randomized in a 1:1 ratio stratified by service or team to undertake training in MATCH at the start or at the completion of the study; and second, young people and their families were randomized in a 1:1 ratio stratified by sex and ethnicity (Māori, Pacific, or an other ethnicity) to receive MATCH or UC. The major ethnic groups in New Zealand include Māori, the Indigenous people of New Zealand (14.9% of the population), New Zealand European people (74% of the population), Asian people (11.8% of the population), and non-Māori Pacific people (7.4% of the population) who have settled in New Zealand from the Pacific Islands such as Samoa. Inequities in the health and mental health for Māori and Pacific people have led to specific mental health services being set up in some regions to attempt to reduce the inequities. However, many Māori and Pacific young people are seen in the mainstream CAMHS.

Electronically generated randomization sequences ensured allocation concealment. Young people, their families, and the research assistants collecting data were blind to allocation.

### Participants

#### Inclusion and Exclusion Criteria for the Clinicians

Clinicians were eligible if they provided clinical treatment to young people and their families at participating CAMHS and provided written, informed consent. They were invited to take part in the study by their service manager.

#### Inclusion and Exclusion Criteria for the Young People

English-speaking young people, aged 7 to 14 years, referred to CAMHS with a primary presenting problem that included anxiety, depression, trauma-related symptoms, or disruptive behavior were eligible for the study. Families were invited to take part in the study by the intake clinician and were included if parents provided written, informed consent and the young person assented. Youth were excluded if they were already being treated, the primary focus of treatment was for another disorder or problem, or a sibling had already been recruited into the study.

### Setting

CAMHS provide mental health care for young people aged 0 to 19 years.^10^ CAMHS are organized into multidisciplinary teams of registered health practitioners, primarily nurses with training in mental health and social workers.^11^

### Interventions

#### MATCH

MATCH is a manualized program of 33 modules addressing 4 problem domains commonly encountered in clinical practice.^12^ MATCH combines written resources, a framework for choice of intervention, and guidance from an online system for monitoring progress and providing timely feedback to clinicians. It combines empirically supported elements of existing therapies within 1 protocol and accommodates comorbidity and changes in clinical presentation during therapy.^13^

Therapists randomized to deliver MATCH were provided with 5 days’ training and then 1-hour weekly Skype-based group consultation (mean [SD] group size, 4 [1.4] individuals; range, 2-6 individuals) provided by MATCH experts (A.M.U., S.K.B., and J.H.). At the start of treatment, the young person and their family collaboratively established the top problems to be addressed.^14^ Clinicians used these problems to tailor treatment. Pharmacotherapy was used as part of standard CAMHS practice, as in UC.

#### Usual Care

Usual care includes case management, psychological therapies, and pharmacotherapy. UC is overseen in multidisciplinary team meetings, typically focused on brief reviews and care coordination.

### Outcomes

Demographic information was provided by parents at enrollment in the study. Because of disparities in mental health outcomes for Māori and Pacific people in New Zealand, ethnicity data were collected.

Research assistants who were blinded to participant treatment group collected most clinical measures by telephone. Additional data were collected from clinicians after the participants had been discharged.

#### Primary Outcomes

In keeping with the primary hypotheses, there were 3 primary outcomes. First, the trajectory of change of clinical severity was assessed using the parent-rated Brief Problem Monitor (BPM)^15^ administered weekly. Second, the fidelity (ie, adherence and competence) with which therapists used EST content^12^ was measured from audio recordings of therapy sessions using the methods and coding system adapted from those used in the initial trial of MATCH.^16^ Therapist competence was rated as follows: 0, not at all; 1, superficial or incomplete; 2, adequate but not optimal; 3, thorough; and 4, expert. Ten percent of recorded therapy sessions (MATCH and UC) were assessed by the research coding team (blind to treatment group). Ten percent of this sample were independently double-coded and had acceptable interrater agreement (mean intraclass correlation coefficient [ICC] on adherence, 0.70; mean ICC on competence, 0.67). Because the results of our initial coding of therapy sessions of UC were markedly different from those of previous studies,^6,7^ we conducted a second round of coding with a subset of 100 randomly selected sessions, coded by experienced independent coders from the US, with 20% of this sample independently assessed by a coder in New Zealand to check for systematic discrepancies between countries. The interrater agreement was acceptable across US and New Zealand coders for adherence (mean ICC, 0.74) and competence (mean ICC, 0.73). Third, the efficiency of service delivery (extracted from logs completed by clinicians) was assessed using duration of therapy (days), clinician time (minutes), and the number of therapy sessions attended and missed.

#### Secondary Outcomes

Secondary outcomes included the youth-rated BPM administered weekly,^15^ the parent- and youth-rated Strengths and Difficulties Questionnaire administered monthly,^17^ the parent- and youth-rated Top Problems Assessment administered weekly,^14^ the Child Health Utility (a quality of life assessment administered at baseline, discharge, and follow-up),^18^ and the number and type of diagnoses assessed at baseline and discharge using the Development and Well-Being Assessment.^19^ Prescribed medications were recorded at baseline, discharge, and follow-up. Clinician satisfaction with therapy was assessed using the Therapist Satisfaction Index.^20^ A treatment satisfaction questionnaire for parents and youth was developed for this study.

### Measures of Harm

Reports of serious adverse events^9^ were collected and reported to the Data Monitoring Committee. At the request of the Data Monitoring Committee, we developed a measure for moderate adverse events that were reported during the study and collected from the parents through specific enquiry at follow-up.

### Sample Size and Power

Details of the initial sample size and changes to it have been published elsewhere.^9^ From the results of previous studies,^7^ we estimated that 200 participants would be needed for 90% power to detect a significant difference in the change that was clinically important (ie, >2 units, with an effect size of approximately 0.45) on the parent BPM change (2-tailed α = .05).

### Statistical Analysis

The primary analyses used the intention-to-treat population. Because neither MATCH nor UC has a fixed duration, testing of the primary clinical hypothesis compared the trajectory of change across time on the parent-rated BPM (total score) as per the original studies.^6,7^ A mixed-effects regression model was used, with outcome = *a*_0_(intercept) + *a*_1_(treatment group) + *a*_2_(time) + *a*_3_(treatment × time), with treatment and time (log_e_ day) treated as fixed effects and the participant intercepts and slopes as random events. Sensitivity analyses were performed to test for the consistency of treatment response across medication use, clinician site, and previous evidence-based therapy training categories, by testing the appropriate interaction terms. Per-protocol analyses included participants who completed the study, received therapy as per their allocated treatment group, and had completed at least 4 therapy sessions. For the second and third primary hypotheses and all treatment satisfaction measures, between-group comparisons were made using univariable ANOVA. Trajectories of change for secondary clinical outcomes were compared for the Strengths and Difficulties Questionnaire and Top Problems Assessment as described previously in this article. Changes in the Child Health Utility were compared between groups using univariable ANOVA to assess changes between baseline, discharge, and at 3-month follow-up. Two-tailed *P* < .05 was considered to indicate statistical significance. Data analysis was performed using SPSS statistical software version 25 (IBM Corp) from April 2016 to July 2017.

## Results

### Group Characteristics

Sixty-five clinicians (mean age, 38.7 years; range, 23.0-64.0 years; 54 female [83%]; 29 New Zealand European [44.6%], 11 Māori [16.9%], 6 Pacific [9.2%], 2 Asian [3.1%], and 17 other 17 [26.2%], including 5 British, 4 American, and 3 South African) were recruited and randomized to receive MATCH training (32 clinicians) or to deliver UC (33 clinicians). They were balanced with regard to age, sex, and ethnicity.

Clinicians included 19 nurses trained in mental health (29.2%), 19 social workers (29.2%), 12 clinical psychologists (18.5%), 11 occupational therapists (16.9%), and 4 other clinicians (6.2%). They had been in their current CAMHS roles for a mean (SD) of 3.5 (3.3) years (range, <3 months to 15 years). Overall clinical practice experience ranged from less than 1 year to 40 years (mean [SD], 11.4 [9.4] years). A similar percentage between groups had previously received training in relevant ESTs, such as cognitive behavioral therapy or parent management training (MATCH, 12 clinicians [38%]; UC, 11 clinicians [33%]) and had at least 10 years postqualification practice (MATCH, 15 clinicians [47%]; UC, 15 clinicians [45%]).

Two hundred six young people (mean age, 11.2 years; range 7.0-14.0 years; 122 female [61%]) (Table 1), were recruited and randomized, with 6 young people (5 from MATCH, 1 from UC) leaving the study before treatment began or any data were collected. Outcome data for at least 2 time points were available for the remaining 200 participants (97%), and 164 participants (80%) completed the 3-month follow-up assessment (Figure).

**Table 1.** Baseline Characteristics by Treatment Group

Characteristic	Participants, No. (%)	
	MATCH (n = 97)	Usual care (n = 103)
Age at study entry, mean (SD), y	10.9 (2.3)	11.3 (2.4)
Sex		
Female	59 (60.8)	63 (61.2)
Male	38 (39.2)	40 (38.8)
Ethnicity (total response)^a^		
New Zealand European or European	84 (86.6)	91 (88.3)
Māori	16 (16.5)	21 (20.4)
Pacific	10 (10.3)	9 (8.7)
Asian	3 (3.1)	3 (2.9)
Other^b^	3 (3.1)	2 (1.9)
Previous use of mental health services?		
Yes	30 (30.9)	48 (46.6)
No	64 (66.0)	53 (51.5)
Missing data	3 (3.1)	2 (1.9)
Service type		
Mainstream child and adolescent mental health services	85 (87.6)	90 (87.4)
Kaupapa Māori Services	6 (6.2)	6 (5.8)
Pasifika Services	6 (6.2)	7 (6.8)
Baseline Brief Problem Monitor, mean (SD), total score (range 0-38)		
Parent	17.9 (7.3)	16.0 (8.0)
Youth	13.5 (6.3)	13.8 (7.0)
Baseline Strengths and Difficulties Questionnaire, mean (SD), total difficulties score (range 0-40)		
Parent	18.9 (6.2)	18.1 (7.0)
Youth	16.6 (6.7)	16.9 (6.5)

Abbreviation: MATCH, Modular Approach to Therapy for Children.

^a^ Ethnicity is recorded as total response; therefore, more than 1 ethnicity may be reported.

^b^ Responses for other ethnicity were 1 Chilean, 1 Iranian, 1 Jamaican, and 2 not specified.

**Figure.**
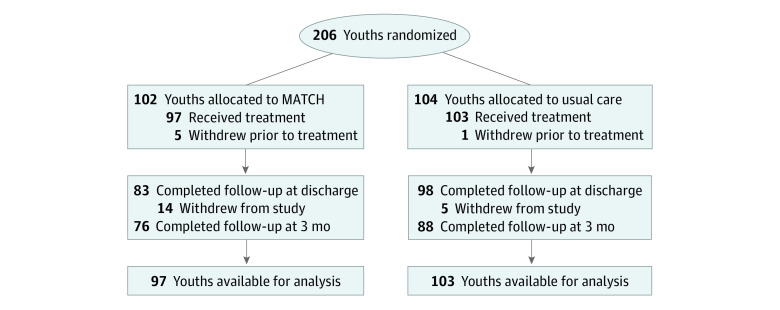
Flow Diagram of Youth Participants MATCH indicates Modular Approach to Therapy for Children.

The 2 groups of youth participants (MATCH and UC) were balanced with regard to age, sex, and ethnicity (Table 1). The ethnic make-up was similar to the general New Zealand population, apart from an underrepresentation of Asian people (Table 1 and Table 2; eTable 1 in Supplement 2).

**Table 2.** Diagnoses by Category at Baseline by Treatment Group

Diagnosis^a^	MATCH		Usual Care	
	Valid, %	Participants, No. (%)	Valid, %	Participants, No. (%)
Depressive disorder	58	14 (24.1)	73	13 (17.8)
Anxiety disorder or posttraumatic stress disorder^b^	61	30 (49.2)	76	49 (64.4)
Disruptive behavior disorder^c^	51	19 (37.2)	71	19 (26.7)
Other^d^	53	7 (13.2)	74	12 (16.3)

Abbreviation: MATCH, Modular Approach to Therapy for Children.

^a^ Diagnoses were made with the Development and Well-Being Assessment. The presence of disorder was defined as less than 50% probability or 50% probability or more.

^b^ Includes separation anxiety, specific phobia, social phobia, panic, agoraphobia, generalized anxiety, posttraumatic stress disorder, and obsessive-compulsive disorder.

^c^ Includes oppositional or conduct disorders.

^d^ Includes autism spectrum disorder, tics, self-harm, bipolar disorder, hyperactivity, and anorexia or bulimia.

### Clinical Outcomes

There were no significant differences between groups on the primary outcome measure, the trajectory of change for total difficulties on the BPM as reported by parents (mean [SE] slope, –1.04 [0.14] and 1-year change of −6.12 in the MATCH group vs –1.04 [0.10] and 1-year change of −6.17 in the UC group; effect size, 0.00; 95% CI, – 0.27 to – 0.28; *P* = .96) and as reported by youths (mean [SE] slope, –0.74 [0.15] and 1-year change of −4.35 in the MATCH group vs –0.73 [0.10] and 1-year change of −4.32 in the UC group; effect size, −0.02; 95% CI, −0.30 to 0.26; *P* = .97) or any other clinical outcome measure (Table 3). Both groups improved significantly, with effects maintained to 3 months of follow-up (eTable 2 in Supplement 2). Sensitivity analyses on the primary clinical outcome showed that the result was not affected by medication use at baseline (*F*_1,183_ = 0.248; *P* = .78), prior relevant EST accreditation of the clinician (*F*_1,183_ = 0.296; *P* = .14), or study site (*F*_1,184_ = 0.483; *P* = .94). There were also no significant differences in the per-protocol analysis on the primary clinical outcome (72 participants in the MATCH group vs 84 participants in the UC group).

**Table 3.** Clinical Outcomes: Trajectories of Change by Treatment Group^a^

Outcome	MATCH (n = 97)		Usual care (n = 103)		Effect size (95% CI)	*P* value
	Slope, mean (SE)	1-Year change	Slope, mean (SE)	1-Year change		
BPM, total						
Parent	–1.04 (0.14)	–6.12	–1.04 (0.10)	–6.17	0.00 (–0.27 to 0.28)	.96
Youth	–0.74 (0.15)	–4.35	–0.73 (0.10)	–4.32	–0.02 (–0.30 to 0.26)	.97
BPM, internalizing						
Parent	–0.66 (0.09)	–3.87	–0.63 (0.07)	–3.71	0.04 (–0.24 to 0.32)	.78
Youth	–0.46 (0.09)	–2.71	–0.38 (0.06)	–2.23	0.13 (–0.15 to 0.41)	.37
BPM, externalizing						
Parent	–0.38 (0.08)	–2.24	–0.41 (0.06)	–2.42	–0.05 (–0.33 to 0.23)	.72
Youth	–0.27 (0.08)	–1.62	–0.36 (0.05)	–2.10		.30
SDQ, total difficulties						
Parent	–1.17 (0.15)	–6.92	–1.17 (0.11)	–6.88	0.01 (–0.27 to 0.29)	.96
Youth	–0.89 (0.16)	–5.27	–1.09 (0.11)	–6.46	–0.17 (–0.45 to 0.11)	.22
SDQ, internalizing						
Parent	–0.70 (0.11)	–4.11	–0.73 (0.07)	–4.33	–0.05 (–0.33 to 0.23)	.72
Youth	–0.50 (0.10)	–2.92	–0.59 (0.07)	–3.48	–0.14 (–0.42 to 0.14)	.33
SDQ, externalizing						
Parent	–0.46 (0.08)	–2.70	–0.42 (0.06)	–2.50	0.06 (–0.22 to 0.34)	.69
Youth	–0.38 (0.10)	–2.25	–0.49 (0.07)	–2.90	–0.16 (–0.44 to 0.12)	.26
Top problems assessment						
Parent	–0.85 (0.09)	–5.03	–0.81 (0.06)	–4.77	0.07 (–0.21 to 0.35)	.61
Youth	–0.94 (0.10)	–5.56	–0.82 (0.07)	–4.84	0.17 (–0.11 to 0.45)	.24

Abbreviations: BPM, Brief Problem Monitor; MATCH, Modular Approach to Therapy for Children; SDQ, Strengths and Difficulties Questionnaire.

^a^ The slope is the estimate of the change in scale score per log day, and the 1-year change is the estimate of the change in scale score 1 year after the initial assessment. The primary clinical outcome was the trajectory of change of parent BPM.

There were no significant differences between groups in the number of diagnoses from before to after treatment (eTable 3 in Supplement 2). Prescription of medications for psychiatric conditions did not differ between treatment groups at baseline or during therapy (eTable 4 in Supplement 2).

### Delivery of EST

EST content adherence was significantly higher in the MATCH group (mean [SD] level of adherence, 80.0% [20.0%] for 58 coded sessions) than in the UC group (mean [SD] level of adherence, 57.0% [32.0%] for 51 coded sessions) (*F*_1,108_ = 23.0; *P* < .001). Therapist competence ratings in delivery of EST content were also significantly higher in the MATCH group (mean [SD], 2.30 [0.57]) than in the UC group (mean [SD], 1.75 [0.50]) (*F*_1,108_ = 8.0; *P* = .001), with both adherence and competence corresponding most closely to an adequate but not optimal rating.^10^ The second round of coding showed almost identical findings, with greater adherence in the MATCH group (58 participants; mean [SD] level of adherence, 81.0% [22.1%]) than in the UC group (42 participants; mean [SD] level of adherence, 56.0% [30.2%]) (*F*_1,76_ = 10.7; *P* = .002) and greater therapist competence in the MATCH group (mean [SD] rating, 2.25 [0.62]) compared with the UC group (mean [SD] rating, 1.76 [0.64]) (*F*_1,79_ = 9.1; *P* = .003).

### Efficiency of Service Delivery

Participants receiving MATCH attended significantly more therapy sessions than did participants in the UC group (mean [SD], 13.4 [8.4] sessions vs 10.7 [7.7] sessions; *F*_1,190_ = 5.6; *P* = .02). However, there were no significant differences in total face-to-face clinician time (mean [SD], 806 [527] minutes in the MATCH group vs 677 [539] minutes in the UC group) or the overall duration of therapy (mean [SD], 167 [107] days in the MATCH group vs 159 [107] days in the UC group) (Table 4).

**Table 4.** Service Delivery Outcomes by Treatment Group

Outcome	MATCH (n = 97)		Usual care (n = 103)		Effect size	*P* Value
	Valid, %	Mean (SD)	Valid, %	Mean (SD)		
Clinician time, min	91	806 (527)	99	677 (539)	0.24	.10
Duration of contact, d	90	167 (107)	100	159 (107)	0.07	.61
Attended therapy sessions, No.	92	13.4 (8.4)	100	10.7 (7.7)	0.34	.02
Missed therapy sessions, No.	92	2.7 (3.0)	100	2.2 (3.4)	0.16	.28

Abbreviation: MATCH, Modular Approach to Therapy for Children.

### Satisfaction With Treatment

MATCH therapists were significantly more satisfied with the treatment they had provided (mean [SD] Therapist Satisfaction Index total scores, 4.0 [0.6] for MATCH delivered to 79 participants vs 3.7 [0.6] for UC delivered to 88 participants; *F*_1,162_ = 9.567; *P* = .002). Parent-rated and youth-rated total satisfaction scores (maximum of 32 and 20, respectively) were high and not significantly different between treatment groups (mean [SD] scores for parents, 25.7 [6.5] for MATCH vs 24.3 [7.3] for UC; *F*_1,170_ = 1.780; *P* = .19; mean [SD] scores for youth, 16.4 [3.7] for MATCH vs 15.5 [4.2] for UC; *F*_1,158_ = 2.076; *P* = .16).

### Harms

There were no significant group differences in terms of serious and moderate adverse events (eTable 5 in Supplement 2). Initial treatment focus by group is shown in eTable 6 in Supplement 2.

## Discussion

Training in MATCH resulted in significantly improved delivery of ESTs by clinicians, and the trajectory of change in clinical outcomes resembled that found in other trials of MATCH.^6,7^ However, the increased delivery of EST did not translate into improved clinical outcomes or efficiency. Young people in both treatment groups improved similarly at discharge and maintained this improvement at 3 months’ follow-up. Effects were not moderated by individual services, clinician training, medication use, or initial focus of treatment.

In 2 previous RCTs,^6,7^ MATCH improved clinical outcomes and service efficiency, increased EST use compared with UC, and increased use of standard manual-based behavioral and cognitive behavioral therapy.^7^ It is notable that similar clinical change was achieved in our study despite fewer therapy sessions and shorter duration than the previous studies. Our study was adequately powered and had more participants per group (200 participants across 2 groups) than the other 2 studies, with 174 participants across 3 groups^7^ and 138 participants across 2 groups.^6^ The training and weekly consultation in all 3 studies was provided by experts from the developers’ team. Despite the use of group rather than individual consultation, the delivery of EST (80.0% adherence in the present study compared with 83% in a previous study^7^) and the trajectory of clinical change in the MATCH group (−1.04 in the current study compared with −0.94 in a previous study^7^) was as good as that achieved in the first trial of MATCH.^7^ The populations studied, clinical severity and context, and measures used in the 3 studies were very similar. The main difference between the studies lies in the extent of EST use in the UC groups, which was 7% to 8% in both previous studies^6,7^ compared with 57.0% in this study. In addition, the trajectory of change in our UC group was equivalent to that in the MATCH group, in contrast to the flatter trajectory in UC in the other 2 studies.^6,7^ An additional RCT comparing MATCH with UC has recently been published^21^ and found delivery rates of EST of 67% for MATCH clinicians and 27% for UC clinicians, but there were no differences between MATCH and UC for any clinical measure. Perhaps 57.0% adherence to ESTs results in clinical outcomes similar to those for 80.0% adherence, whereas 7% to 8%, as reported in the US,^6,7^ does not. The most recent trial of MATCH^22^ also showed MATCH clinicians providing higher levels of EST than UC but no differences in clinical outcomes.

Training in MATCH is consistently associated with improved levels of EST delivered, whereas the variation in level of EST provided in UC across the 4 RCTs is marked. We considered the possibility of contamination between MATCH and UC, with UC clinicians learning MATCH techniques through team meetings that are the norm in New Zealand. However, case discussions in team meetings are not detailed, and measures to protect against contamination in the current study were identical to those in previous studies.^6,7^ The intensive monitoring involved in this study that may have improved UC is also common to all studies, and so is unlikely to explain the differences. The efforts undertaken to upskill the workforce through training in individual ESTs over the last 2 decades may have been successful in improving UC in New Zealand.

### Strengths and Limitations

Strengths of this study include the multisite clinical settings to maximize generalizability, measurement of adverse events and clinical changes, allocation concealment, and blinded assessments and analyses.^9^ Limitations include the underrepresentation of Asian young people, the single-blind nature of the study, and the low percentage of Development and Well-Being Assessment completion. The ICC ratings for the coding of ESTs were lower than we would have liked. There was no clear pattern of disagreement, although the sample of sessions used for coding fidelity, which was appropriate for assessing overall intercoder reliability of the system within New Zealand and between New Zealand and US coders, was not large enough to do more fine-grained analyses.

## Conclusions

These findings suggest that a brief 5-day training in MATCH resulted in a significant increase in the delivery of ESTs. The lack of change in clinical outcome in our study despite a significant change in delivery of ESTs and in contrast to previous studies may be partly explained by the high level of EST delivered by UC clinicians. The question of how EST fidelity is related to clinical outcomes, and the part played by nonspecific factors such as warmth and empathy, warrants further attention.

## Notes

Supplement 1. **Trial Protocol**

Supplement 2. **eTable 1.** Individual Diagnoses at Baseline by Treatment Group **eTable 2.** Clinical Outcomes: Baseline, Post-Intervention and Follow-up Scores—Intention-to-Treat Analyses **eTable 3.** Changes in Individual Diagnoses by Treatment Group **eTable 4.** Psychotropic Medication Use by Treatment Arm **eTable 5.** Adverse Events by Treatment Group **eTable 6.** Initial Treatment Focus by Treatment Group

Supplement 3. **Data Sharing Statement**
