# Supplementary material for: Effect of Clinician Training in the Modular Approach to Therapy for Children vs Usual Care on Clinical Outcomes and Use of Empirically Supported Treatments: A Randomized Clinical Trial
Source: JAMA Netw Open. 2020 Aug 17;3(8):e2011799. doi: 10.1001/jamanetworkopen.2020.11799 (PMC7431993; doi:10.1001/jamanetworkopen.2020.11799)
Supplement: Supplement 1. — Trial Protocol [file jamanetwopen-3-e2011799-s001.pdf]

Treatment Approaches for Children and Young people (TrACY) in CAMHS Study

STUDY PROTOCOL

Protocol title	Treatment Approaches for Children and Young people (TrACY) in Child and Adolescent Mental Health Services (CAMHS) Study
Principal investigators	**Professor Sally Merry and Dr Sue Crengle**<br>**c/- Department of Psychological Medicine, F.M.H.S., Private Bag 92019, Auckland, New Zealand 1142**<br>**Phone +64 9 373 7599 ext 86981**
Study consultant	**Professor John Weisz**
Co-investigators	**Dr Karolina Stasiak, Dr Mathijs Lucassen, Dr Ainsleigh Cribb-Su'a, Dr Jik Loy, Associate Professor Chris Frampton, Assistant Professor Sarah Kate Bearman, Dr Ana Ugueto, Dr Jenny Herren and Dr Monique Faleafa.**
Study manager	**Dr Mathijs Lucassen (1 Oct 2013 - 1 June 2015)**<br>**Sarah Hopkins (1 June 2015 – 30 Sept 2016)**<br>**Department of Psychological Medicine, F.M.H.S., Private Bag 92019, Auckland, New Zealand 1142**<br>**Phone +64 9 373 7599 ext 82015**
Reviewed & funded by	**The Health Research Council (New Zealand)**
Date	**Original protocol: 3 July 2013 (updated 5 February 2016 – v4)**

Table of contents

Table of contents 2
List of abbreviations 3
1. Overview 4
2. Rationale 8
3. Flowchart and timeline 14
4. Study methods 16
5. Administration 28
6. References 31

List of abbreviations

BPM	Brief Problem Monitor
CAMHS	Child and Adolescent Mental Health Service
CHU9D	Child Health Utility Measure
DAWBA	Development and Well-Being Assessment
DHB	District Health Board
EBT	Evidence-Based Therapy/Treatments
HoNOSCA	Health of the Nations Outcome Scale for Children and Adolescents
MATCH-ADTC or MATCH	Modular Approach to Therapy for Children with Anxiety, Depression, Trauma and Conduct problems
SDQ	Strengths and Difficulties Questionnaire
TPA	Top Problem Assessment
TrACY	Treatment Approaches for Children and Young people
TSI	Therapist Satisfaction Inventory
UC	Usual Care

1. Overview

1.1 Short title: The TrACY Study

1.2 Investigators and study centres: The overall design and conduct of this trial is the responsibility of the co-principal investigators, study consultant, co-investigators and trial steering committee. The study will be based at the Department of Psychological Medicine, University of Auckland. Teams from several District Health Board (DHB) Child and Adolescent Mental Health Services (CAMHS) will enrol participants in the study.

1.3 Study period: 1 October 2013 to 30 September 2016 (study set up, clinician training, recruitment, data analysis, write up and dissemination of results). Participant recruitment is expected to begin in March 2014.

1.4 Objective: To improve the overall quality of care received by children and adolescents attending child and adolescent mental health services (CAMHS) in New Zealand. Specifically for those children or adolescents with anxiety, depression, trauma-related symptoms and disruptive behaviour – for which MATCH-ADTC (Modular Approach to Therapy for Children with Anxiety, Depression, Trauma and Conduct problems) has been developed and formally evaluated with.

1.5 Study design and methodology: This is a pragmatic multi-site randomised controlled effectiveness trial comparing MATCH to usual care (UC). Participants will be clinicians from participating CAMHS and child/adolescent participants (and their parents/caregivers) referred to CAMHS.

1.6 Study population: Children and adolescents 7 to 14 years old with anxiety, depression, trauma and disruptive behaviour of a severity that warrants treatment in a CAMHS.

1.7 Number of participants: Up to 400 child participants will be recruited (200 in each arm). Up to 60 clinicians will be recruited to deliver the treatments (i.e. either MATCH or UC). We aim to recruit 100 Māori and 100 Pacific child participants as part of the overall group.

> **1.7 Amendment (June 2015):** Sample size reduced to at least 200 child participants (approximately 100 in each arm).
>
> **Notes:**
>
> - Reasons for reduced recruitment:
>   - Our recruitment rate within mainstream services has mostly been in line with our original estimations (that is, approximately 8 participants per clinician).
>   - However, clinician attrition across all study sites was 50% higher than our estimations (34% attrition) and likely reflects the general turnover of staff at CAMHS sites.

  - Reduced ability to recruit at Kaupapa Māori and Pacific clinics was due to high resource pressure at services, higher acuity of potential participants, fewer referrals of participants in the eligible age range and logistic restrictions placed upon the selection and number of clinicians able to participate in the study.
- Due to budgetary restrictions and logistic considerations at the study sites, it was not possible to extend recruitment past June 30th 2015.
- The recruitment of participants from Māori and Pacific providers has fallen well short of our original study aims. Consequently, sub-analyses will no longer be conducted for our ethnic groups.
- A sample size of 200 participants will provide sufficient power to detect a meaningful difference in our primary outcome measure among the entire cohort.

1.8 Main criteria for inclusion: Children and adolescents will be eligible for inclusion in the trial if:

- They are newly referred to CAMHS with a primary disorder that includes anxiety, depression, trauma-related symptoms or disruptive behaviour;
- They are 7 to 14 years of age on the date of consent;
- They are able to provide written consent (or verbal assent) and have written parental/guardian consent; and,
- The child and their parent/guardian can speak English or there is a clinician who can provide the necessary treatment, according to the treatment arm the participant is randomised to, in the family's native language.

1.9 Main criteria for exclusion: Children and adolescent will be ineligible for inclusion in the trial if:

- They are currently receiving other treatment for their disorder from the CAMHS (or another service); or,
- They have a primary disorder of psychosis, severe intellectual disability, attention deficit-hyperactivity disorder (where the primary reason for referral is inattention and/or over-activity), autism or other pervasive developmental disorder, anorexia nervosa or bulimia nervosa; or,
- The young person is acutely suicidal; or,
- They have a sibling that has previously been recruited into the study.

1.10 Intervention: Child and adolescent participants will receive MATCH therapy from a CAMHS clinician (who usually provides clinical interventions in CAMHS) who has received MATCH training from the MATCH trainers from the USA (and the clinician will be engaged in weekly MATCH consultation sessions with MATCH trainers after the initial face-to-face training). In summary, MATCH provides effective elements of the evidence-based treatments (EBTs) for child anxiety, depression, trauma-related symptoms and disruptive behaviour in one protocol; it caters for co-morbidity and provides an opportunity to address different disorders that may emerge during therapy. MATCH is comprised of 33 modules (i.e. specific treatment procedures) which can be organised in a flexible manner.

1.11 Duration of treatment: Duration of treatment will vary, based on the need of the client and service-specific constraints (e.g. the need to treat rapidly in order to meet the demands of the service).

1.12 Control: Child and adolescent participants will receive UC from a CAMHS clinician.

1.13 Criteria for evaluation:

Primary outcome measures

- The primary clinical outcome measure is The Brief Problem Monitor (BPM) [see Primary Hypothesis 1].
- The primary EBT outcome measure is based on audio-recordings of all therapy sessions (a random sub-set (approximately 5 to 10%) will be analysed from both the MATCH and UC treatment arms and analysed for content of EBT) [see Primary Hypothesis 2].
- The primary efficiency of services outcome measure is based on total therapy input by clinicians (in minutes), contact with the CAMHS (in weeks), number of therapy sessions and number of missed therapy sessions [see Primary Hypothesis 3].

Secondary outcomes

- The Strengths and Difficulties Questionnaire (SDQ);
- Top Problems Assessment (TPA) (which will be administered by telephone) to be collected weekly and at follow-up;
- Diagnoses using the Development and Well-Being Assessment (DAWBA) at baseline and after discharge from CAMHS;
- The Child Health Utility (CHU9D) measure at baseline, after discharge and at follow-up.

Other measures

- Treatment provided in UC;
- Child's/adolescent's psychotropic medication use;
- Clinician rated serious adverse events;
- The Therapist Satisfaction Inventory (TSI) (to determine clinicians' satisfaction with therapy);
- Child/adolescent survey of treatment satisfaction;
- Parent/caregiver survey of treatment satisfaction; and,
- CAMHS manager/team leader satisfaction of MATCH-ADTC.

1.14 Statistical methods:

Study power: Approximately 8 child or adolescent participants per clinician will be needed (400 child or adolescent participants in total) in order to detect moderate effect sizes. In total, 50 clinicians (we will recruit up to 60 clinicians to allow for approximately 15% attrition) will provide sufficient power (80%) to detect effect sizes of approximately 0.37 as statistically significant (two-tailed $\alpha=0.05$) for the comparison of outcome measures. We plan to have approximately 12 clinicians from Māori services and 12 clinicians from Pacific services.

Independent analyses undertaken for each of these services will, therefore, have sufficient power (80%) to detect moderate effect sizes of approximately 0.61 to 0.68 as statistically significant.

1.14 Revised study power: Because of the difficulties with recruitment, a revised power calculation was carried out in January 2015. By treating the whole group as a single cohort, a sample size of 200 (100 per treatment arm) will provide sufficient power (80%) to detect moderate effect sizes of approximately 0.5 to 0.55 as statistically significant (two-tailed $\alpha=0.05$) for the comparison of outcome measures. These calculations are based on interim analyses of the first 45 completed cases and use a valid estimation of overall variability and acknowledgement of a meaningful change in the primary outcome measure (a different of at least 2 points on externalising and internalising subscales of the BPM).

Statistical analyses: Both per-protocol and intention-to-treat analyses will be carried out. Both treatments (i.e. MATCH and UC) will vary in content and duration, so primary analyses will be conducted using trajectories of change across time on the Brief Problem Monitor (BPM) as the primary outcome measure.

1.15 Funding: The Health Research Council of New Zealand.

2. Rationale

2.1 What is the problem?

- Mental health problems are common in children and adolescents (1-5), the impact is considerable and it has been estimated that 50% of all adult mental health disorders have their onset in adolescence (6).
- There are a number of evidence-based treatments (EBTs) for the most common mental health problems, namely anxiety, depression, trauma-related symptoms and disruptive behaviour (1). The Ministry of Health in New Zealand has repeatedly asserted the need for EBTs in mental health generally (7-9), and in CAMHS specifically (9). Cognitive behavioural therapy is an effective intervention for anxiety (46 randomised controlled trials/ RCTs), depression (18 RCTs), trauma-related symptoms (6 RCTs), and behavioural parent training is the treatment of choice for disruptive behaviour (32 RCTs) (10-13).
- Although therapies have been shown to be effective in research settings, they are not easily translated into practice (14, 15). This is true internationally and is probably because: a) Traditional EBTs lack flexibility and focus on one disorder (16) and training for each single-focus therapy is time-consuming and costly; b) Research participants in RCTs are typically treated for one disorder whereas in clinical practice co-morbidity is common; and c) Clinical presentations may change during therapy, for example a child with depression may develop disruptive behaviours.
- Clinicians in New Zealand often do not have the training they need to deliver EBTs for the range of problems that present at CAMHS. In New Zealand, CAMHS provide secondary level services that deal with the 3-5% most severe mental health problems of those aged 0-19 years (17). The two largest professional groups in CAMHS are nurses and social workers (18) and their pre-registration courses do not include psychological therapies (19). Therefore, most learning in CAMHS occurs 'on the job'.
- Currently, clinicians have to train in multiple EBTs which is expensive and takes several years. For example, training in CBT is available in New Zealand in a one-year postgraduate course, which can only be accessed by up to 12 clinicians per year. Accredited training in Incredible Years (a form of parent management training) takes a year and can be accessed by up to 35 clinicians per year. Little training is available outside these courses which means that training in EBTs can only reach a small minority of the approximate 1,000 full-time equivalent CAMHS clinicians (18). The extent to which EBTs are currently used in CAMHS in New Zealand is not known, but based on CAMHS clinicians' access to training and from overseas research (20-22), it is likely to be variable across services and at times very limited.
- Services need to deliver EBTs in a manner responsive to Māori and Pacific children. Māori, Pacific and other ethnic minority groups have been shown to have higher rates of mental health problems (23-28) yet are seldom specifically included in RCTs.
- By 2020, there is likely to be a doubling of the demand on mental health services, which is only going to be met by a 30-40% increase in funding. This means that we need a 50-60% lift in productivity which is a huge challenge for the status quo of mental health

services (29). The Health Workforce 2011 Report calls for delivering mental health services more effectively and more productively (29). The report highlights the need to build and up-skill the clinical workforce, to use innovations to leverage scarce workforce resources and to ensure that therapy is delivered efficiently and effectively (29).

2.2 A new flexible efficient modular approach to therapy

Professor John Weisz, from Harvard University, and his team have been part of an initiative to develop a practical EBT system that is designed to work in day-to-day practice across a range of clinical problems. Following a number of meta-analyses which identified the therapies with the best evidence for effectiveness (15, 30, 31) Prof Weisz and his team have developed a unique treatment manual and training package (called MATCH) for children with anxiety, depression, trauma-related symptoms and/or disruptive behaviour. It is specifically designed to combine the effective elements of the EBTs for these problems in one protocol, cater for co-morbidity and provide an opportunity to address different disorders that may emerge during therapy. MATCH is comprised of 33 modules (i.e. specific treatment procedures) which can be organised in a flexible manner. Clinicians are guided by an evidence-based algorithm to tailor treatment to each child's characteristics and needs. Children/young people and their families are also given an integral role in defining the goals of therapy. Clinicians use a web-based system called Treatment Response Assessment for Children (TRAC) to monitor progress and adapt therapy in consultation with a child/young person and their family until a problem is resolved.

MATCH was evaluated in a large RCT (n=174) comparing it against standard (single-focus) EBT and usual care in the United States of America (USA). The study was published in the prestigious *Archives of General Psychiatry*. The results showed that MATCH was significantly more effective than standard EBT and usual care, with effect sizes of 0.59-0.71 on the primary outcome variables (32).

2.3 The need for a trial in New Zealand

MATCH has been developed and evaluated in the USA, but before it can be introduced in New Zealand CAMHS it should be formally tested here. The trial we are proposing will provide New Zealand-specific assessment of effectiveness (clinical outcomes and service efficiencies) and a specific investigation of the acceptability and effectiveness for Māori and Pacific people. New Zealand has a growing child and adolescent population, especially Māori and Pacific population (33, 34), who are at increased risk of mental health problems (23-28).

Our trial will also contribute to the international literature by: (a) Testing the effectiveness of MATCH in publicly funded mental health services (a healthcare delivery model that is different from that in the USA); and (b) Providing information about the effectiveness of MATCH across different ethnic groups.

This study has practical implications for the workforce. All CAMHS clinicians (e.g. nurses, social workers and occupational therapists) should receive training in therapies that address the

common problems that present at services, but training is costly, time-consuming and limited. MATCH is the first comprehensive EBT system that could realistically become part of a 'core competency' training package for all CAMHS clinicians. Training in MATCH takes only six days, followed by one-hour weekly group consultation sessions. Comparable training using traditional EBTs for all the disorders included in MATCH would comprise 17 days of training and 2-3 hours per week of supervision for the duration of training.

2.4 Responsiveness to Māori:

Almost half (44%) of the Māori population is between 0-19 years of age (18). Māori children have higher exposure to environmental factors that increase risk of mental health difficulties, higher rates of mental health problems (23, 35, 36), and account for about 26% of the total clients (0-19 years old) attending CAMHS and Alcohol and Drugs services in New Zealand (18). Between 2008 and 2009 there was a 38% increase in Māori clients accessing CAMHS (18). In 2009, 1.76% of the Māori 0-19 year population accessed care, rates higher than those of the Pacific (0.99%) and Asian (0.46%) populations, and the 'total population' (1.49%) (18). In 2010 86% of Māori children/young people seen in CAMHS accessed 'mainstream' services and 14% accessed Kaupapa Māori services (18). It is, therefore, important to assess whether new therapies that may be introduced into CAMHS are acceptable and efficacious for the Māori children/young people and their whānau who access these services. If MATCH is shown to be acceptable and efficacious for Māori children and whānau, the range of known effective therapies available for Māori will be increased. However, this does not diminish the need for the development of therapies by Māori and for Māori.

MATCH was developed in the United States using the best available evidence about effective treatments for anxiety, depression, trauma-related symptoms and disruptive behaviour in children and adolescents. It provides modules for therapeutic management of these conditions, resources that can be used during therapy and guidelines to assist the clinician to make choices about therapeutic approaches. However, MATCH does not specify how the clinician engages, develops relationships, and communicates with the children and their families. This will allow Kaupapa Māori services and clinicians working with Māori children and whānau to continue to use their culturally appropriate practices and tikanga. Furthermore, we are able to develop culturally appropriate resources for Māori children and whānau that can be used with the MATCH modules.

We have specifically approached and met with Kaupapa Māori services (e.g. He Kaakano at Counties Manukau DHB and Te Whare Marie at Capital and Coast DHB) and they are keen to participate in the trial. One mainstream CAMHS (Te Roopu Kimiora, Northland DHB) has a high proportion of Māori clients (approximately 50%) and this CAMHS has expressed considerable interest in participating in a RCT of MATCH. We are hoping that through recruitment in Kaupapa Māori and mainstream services approximately 130 Māori children/young people will participate in the trial (allowing us to meet our aim of at least 100 Māori children/young people, and increasing the power of our Māori analyses). The study has been designed to ensure power to identify moderate effect sizes in clinical outcome measures for Māori children/young people.

We will also be able to examine service level (process measures) separately for Kaupapa Māori services. We have Māori researchers on the team. Dr Crengle shares the Principal Investigator role with Associate Professor Merry, and Dr Cribb-Su'a will be the Māori New Zealand MATCH advisor. Both Drs Crengle and Cribb-Su'a have been involved in the design of the study since its inception. We have met with an advisory group compromising a kaumātua (Rawiri Wharemate) and Māori clinicians experienced in work with children and adolescents with mental health problems (Dr Matt Shepherd and Tania Cargo – Clinical Psychologists, Janice Beazley – Māori Clinical Advisor from the Werry Centre, Dr Cribb-Su'a and Dr Crengle). After our initial meetings, this group advised us to proceed with the development of a full Health Research Council proposal and has agreed to provide on-going oversight for the duration of the project.

2.4 **Amendment (August 2015):** Our ability to recruit at Kaupapa Māori and Pacific clinics was significantly reduced as a result of resource pressure at services, high acuity of potential participants, fewer referrals of participants in the eligible age range and logistic restrictions placed upon the selection and number of clinicians able to participate in the study. Consequently, we will no longer be conducting sub-analysis for our ethnic groups as reduced recruitment in these groups does not allow for meaningful statistical comparisons. However, we do intend to carry out supplementary qualitative research to establish the views of Māori participants and clinicians regarding the appropriateness of MATCH for these populations. We are working in consultation with the Māori co-investigators in our research team and Māori clinicians within the TrACY study to ensure that this research is carried out in the most appropriate manner.

2.4 **Amendment (February 2016)**: We will supplement our primary analyses with the addition of key informant interviews with Māori and Pacific clinicians who delivered MATCH, as well as their team leaders. Through these key informant interviews, we would like to establish their views regarding the appropriateness of MATCH and the key adaptations for the culturally-sensitive implementation of MATCH for these populations.

Specifically we would like to explore the following with appropriate study clinicians:

1. Their perspective on the acceptability and appropriateness of MATCH for use with Māori/Pasifika children and young people (CYP).
2. The strengths and limitations of MATCH for use with Māori/Pasifika CYP.
3. What, if any, adaptations clinicians made to the way MATCH was used in the therapy session to ensure that it was delivered in a culturally appropriate way?
4. Any recommendations that could be incorporated for use with Māori/Pasifika CYP if MATCH was implemented nationally.

During the TrACY trial the clinicians providing MATCH have received supervision from experienced MATCH therapists who live and work in the USA. We would also like to explore clinician's experience of this supervision.

5. How useful was the MATCH supervision?

6. Were recommendations made during supervision consistent with the kaupapa (philosophy) and tikanga (practices) used with Māori/Pasifika CYP by the clinician/service?

2.5 Responsiveness to Pacific people:

We have purposely approached Pacific CAMHS and invited them to participate in the trial. To date, Vaka Toa (Counties Manukau DHB) and Health Pasifika (Capital and Coast DHB) are keen to participate. We are hoping that through recruitment in Pacific and mainstream services approximately 130 Pacific children/young people will participate in the trial (allowing us to meet our aim of at least 100 Pacific children/young people, and increasing the power of our Pacific analyses). The study has been designed to ensure power to identify moderate effect sizes in clinical outcome measures for Pacific children/young people. We will also be able to examine service level (process measures) separately for Pacific services. We have an experienced Pacific clinician, researcher and workforce development leader on the team, Dr Faleafa, who has been involved in the design of the study since its inception and she has met with Pacific CAMHS with others from the research team. Dr Faleafa will be the Pacific New Zealand MATCH advisor.

2.5 **Amendment (August 2015):** Our ability to recruit at Kaupapa Māori and Pacific clinics was significantly reduced as a result of resource pressure at services, high acuity of potential participants, fewer referrals of participants in the eligible age range and logistic restrictions placed upon the selection and number of clinicians able to participate in the study. Consequently, we will no longer be conducting sub-analysis for our ethnic groups as reduced recruitment in these groups does not allow for meaningful statistical comparisons. However, we do intend to carry out supplementary qualitative research to establish the views of Pacific participants and clinicians regarding the appropriateness of MATCH for these populations. We are working in consultation with the Pacific co-investigators in our research team and Pacific clinicians within the TrACY study to ensure that this research is carried out in the most appropriate manner.

2.5 **Amendment (February 2016)**: We will supplement our primary analyses with the addition of key informant interviews with Māori and Pacific clinicians who delivered MATCH, as well as their team leaders. Through these key informant interviews, we would like to establish their views regarding the appropriateness of MATCH and the key adaptations for the culturally-sensitive implementation of MATCH for these populations.

Specifically we would like to explore the following with appropriate study clinicians:

1. Their perspective on the acceptability and appropriateness of MATCH for use with Māori/Pasifika children and young people (CYP).
2. The strengths and limitations of MATCH for use with Māori/Pasifika CYP.
3. What, if any, adaptations clinicians made to the way MATCH was used in the therapy session to ensure that it was delivered in a culturally appropriate way?

4. Any recommendations that could be incorporated for use with Māori/Pasifika CYP if MATCH was implemented nationally.

During the TrACY trial the clinicians providing MATCH have received supervision from experienced MATCH therapists who live and work in the USA. We would also like to explore clinician's experience of this supervision.

5. How useful was the MATCH supervision?
6. Were recommendations made during supervision consistent with the kaupapa (philosophy) and tikanga (practices) used with Māori/Pasifika CYP by the clinician/service?

3. Flowchart and timeline

3.1 Study flowchart

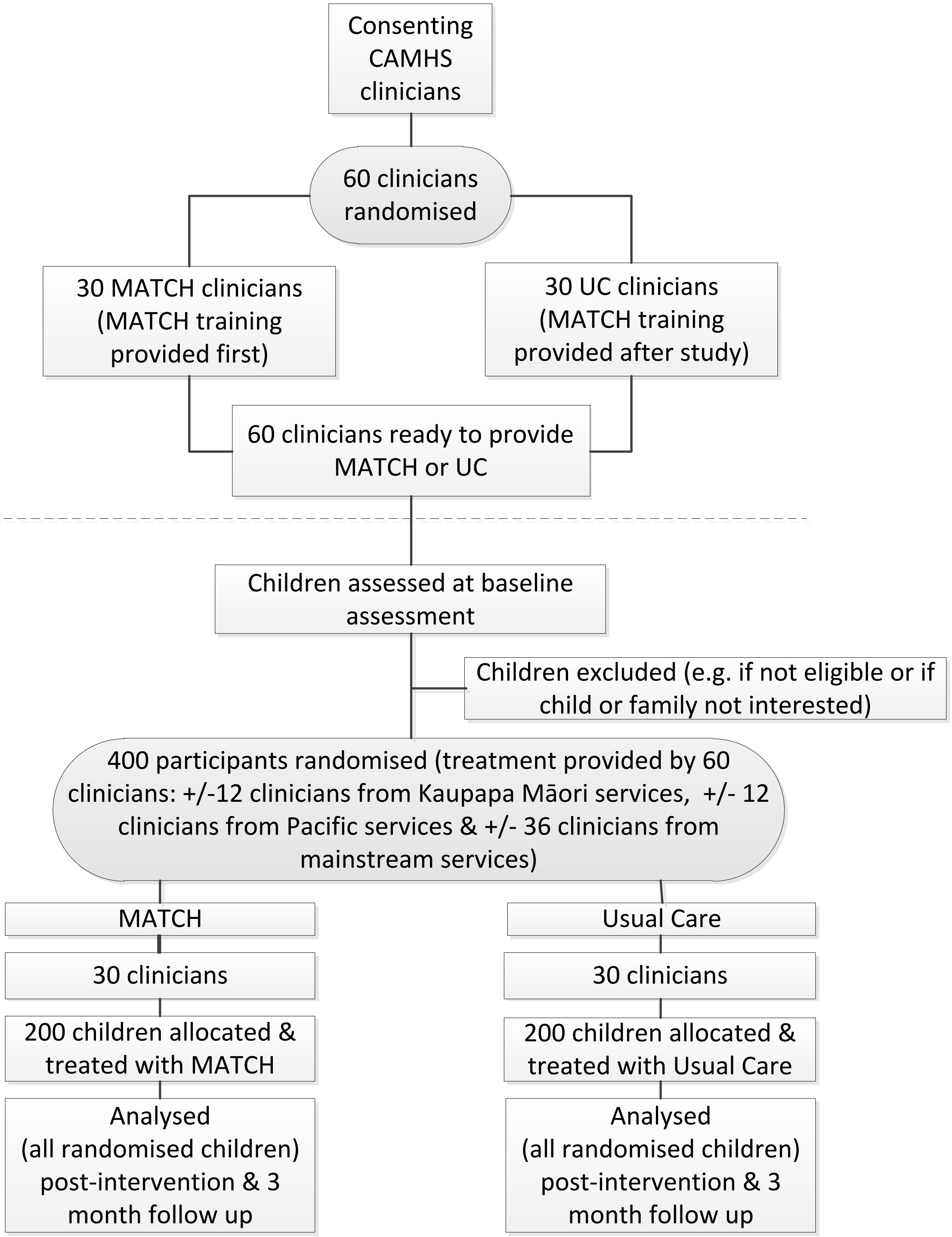

3.2 Study timeline

- July 2013: Ethics application submitted
- Oct 2013: HRC funding starts
- Oct 2012-Jan 2013: Set up study sites and test systems
- Feb 2014: MATCH training (for clinicians randomised to MATCH)
- March 2014: Participant recruitment begins (recruitment for +/- 15 months) (Note: we believe we will be able to recruit within this time period based on information provided by participating services).
- June 2015: All participant recruitment completed
- Jan-Feb 2016: All 3 month follow-up assessments completed with participants, all service use parameters data collected
- End of Mar 2016: Data clean up and lock down
- April to Sept 2016: Data analysis, feedback to sites and community, dissemination to wider academic and other communities, MATCH training for UC clinicians

4. Study methods

Our group is made up of researchers and clinicians with a commitment to improve the overall quality of care received by children and adolescents attending CAMHS in New Zealand, by demonstrating *improved clinical outcomes and increased delivery of EBT*.

We have used Donabedian's framework (a method of assessing the quality of healthcare) of structure, process and outcome (37) to determine the potential impact of delivering MATCH in New Zealand. In this study our focus is on outcomes and process.

4.1 Primary hypotheses:

The primary hypotheses are that training CAMHS clinicians in MATCH compared with UC will:

1. Improve clinical outcomes for children and adolescents accessing CAMHS [*outcomes*] (measured by comparing difference in trajectory of change of clinical severity (i.e. which group improves more quickly));
2. Increase the delivery of evidence-based therapy (EBT) [*process*] (measured by EBT content of audiotaped therapy sessions);
3. Yield equal or better efficiency of service delivery [*process*] (measured by clinician minutes to deliver therapy and duration (in weeks) of contact with the service by children, adolescents and their families).

4.2 Study design:

A pragmatic multi-site randomised controlled effectiveness trial (RCT) comparing MATCH with UC (i.e. the therapies usually used in secondary child and adolescent mental health services).

We intend to recruit approximately 60 clinicians from several CAMHS teams (from various DHBs) to deliver treatment to 400 child or adolescent participants over a 15 month period. Of these 400 participants, approximately one quarter (i.e. 100 participants) will be Māori and one quarter (i.e. 100 participants) will be Pacific.

> **4.2 Amendment (June 2015)**: Sample size reduced to at least 200 participants in the overall group, with no specific Māori or Pacific targets.

4.3 Inclusion and exclusion criteria – child and adolescent participants

Criteria for inclusion: Children and adolescents will be eligible for inclusion in the trial if:

- They are newly referred to a participating CAMHS with a primary disorder that includes anxiety, depression, trauma-related symptoms or disruptive behaviour;
- They are 7 to 14 years of age on the date of consent;
- They are able to provide written consent (or verbal assent) and have written
- parental/guardian consent; and,

- The child and their parent/guardian can speak English or there is a clinician who can provide the necessary treatment, according to the treatment arm the participant is randomised to, in the family's native language.

Exclusion criteria: Children and adolescent will be ineligible for inclusion in the trial if:
- They are currently receiving other treatment for their disorder from the CAMHS (or another service); or,
- They do not have a primary disorder of psychosis, severe intellectual disability, attention deficit-hyperactivity disorder (where the primary reason for referral is inattention and/or over-activity), autism or other pervasive developmental disorder, anorexia nervosa or bulimia nervosa;
- The young person is acutely suicidal; or,
- They have a sibling that has previously been recruited into the study.

4.4 Inclusion and exclusion criteria – CAMHS clinicians

Criteria for inclusion: Clinicians will be eligible for inclusion in the trial if:
- They are involved in the assessment and clinical treatment of children and adolescents and their families at participating CAMHS. We anticipate that most clinicians will be nurses, social workers and psychologists (with some occupational therapists, psychotherapists and medical practitioners).
- The clinician is full-time (or at least 0.6 FTE); and,
- The clinician anticipates working at the CAMHS for at least 15 months after the MATCH training is provided.

Exclusion criteria: Clinicians will be ineligible to inclusion in the trial if:
- They provide non-clinical support, rather than assessment or treatment. For example, cultural advisors or youth workers who provide non-clinical interventions.

4.5 Recruitment:

Recruitment will be via CAMHS clinicians. Eligible participants will be invited to participate by a clinician whom they have met.

4.6 Screening eligibility check:

Potential participants will be identified by CAMHS clinicians during their standard initial assessment (or intake assessment). Those children and adolescents with primary disorder that includes anxiety, depression, trauma-related symptoms or disruptive behaviour aged 7 to 14 years old will be invited to participate.

4.7 Participant information and consent:

Child and adolescent participants and their parents/caregivers will be given written information sheets and consent forms. Written consent will be collected from all parents/caregivers.

Written consent (or verbal assent for younger participants) will be collected from all child or adolescent participants. Consent or assent will be sought from children and adolescents depending on their developmental stage. This will be done on a case-by-case basis and determined by the clinician. Those who do not want to participate will receive the care that is usually provided in that service.

4.8 Baseline assessments:

The following baseline data will be collected before the child/adolescent starts treatment:

- Demographics;
- SDQ;
- BPM;
- TPA;
- DAWBA; and,
- Child Health Utility (CHU9D).

The following information will be collected from the child's/adolescent's CAMHS clinician:

- Child's/adolescent's medication use.

Self-rated assessments will be read out to children and young people who may have difficulty filling out these forms without this assistance.

4.9 Randomisation:

Randomisation will occur on two levels:

- *Randomisation of clinicians:* All clinicians from participating CAMHS teams will be invited to participate. Consenting CAMHS clinicians will be block randomised (by service/team) in a 1:1 ratio to MATCH or UC. The block size will vary across sites depending upon the number of individuals likely to be recruited from each site. Clinicians will be stratified on the basis of previous evidence-based therapy training (i.e. those with versus those without accredited training in cognitive behavioural therapy or behavioural parent training).

- *Randomisation of child and adolescent participants:* After the eligibility check, and once consent/assent and collection of baseline assessments have been completed, participants will be randomised in a 1:1 ratio to receive MATCH or UC stratified by sex and ethnicity.

Allocation concealment will be assured by using centralised computer generation of the randomisation sequence.

We will use web-based randomisation procedures to determine treatment allocation.

4.10 Blinding:

The assessors of the outcome measure/s (i.e. those completing the BPM and DAWBA with participants) will be blind to treatment allocation. Those research assistants administering the assessments (i.e. BPM and DAWBA) will not have access to any data that may unblind them. Due to the nature of the intervention (i.e. MATCH) and UC, it will be obvious to clinicians which treatment they are providing. Assessor blinding will be maintained by ensuring that the research assistants (who will collect assessment data) will be unaware of treatment allocation.

4.11 Study intervention:

MATCH (intervention) consists of a manual and a training package (i.e. six days of block training and a year of weekly telephone/Skype consultation sessions from a MATCH trainer to support clinicians in the use of MATCH). MATCH was specifically designed to combine the effective elements of the EBTs for anxiety, depression, trauma-related symptoms and disruptive behaviour in one protocol, cater for co-morbidity and provide an opportunity to address different disorders that may emerge during therapy. MATCH is comprised of 33 modules/specific treatment procedures which can be organised in a flexible manner. Children/adolescents and their families are also given an integral role in defining the goals of therapy. Clinicians use a web-based system (specifically developed for this study) to monitor progress and adapt therapy (The TrACY eMonitor) in consultation with a child/adolescent and their family until a problem is resolved.

Usual care/UC (control) group: Usual care will be the treatment that is usually provided to a child/adolescent at a CAMHS (e.g. case management, therapeutic group work and psychotherapy). Information on what UC was provided to each child/adolescent participant will be collected. A random sub-set of audio recorded therapy sessions will also be reviewed for content of EBT.

4.12 Outcome measures:

The outcome measures have been chosen carefully to ensure that they have robust psychometric properties and to reduce the burden for children, their families and clinicians.

Primary outcome measures:

<u>Clinical outcome measure.</u> The Brief Problem Monitor (BPM) is based on the Brief Problem Checklist (42) and will be collected weekly. The BPM is a 19-item assessment using data from parents and children to measure internalising, externalising, hyperactivity and total problems. It was developed from the widely used Child Behavior Checklist (CBCL) and Youth Self Report (YSR) (43). The BPM has sound psychometric properties (42). It can be administered by phone or face-to-face and takes approximately five minutes to complete, making it a practical and robust measure of the trajectory of change in clinical symptoms over time.

<u>Delivery of EBT</u>. All MATCH and UC therapy sessions will be audiotaped. A randomly selected subset approximately 5-10 % (both MATCH and UC) will be assessed by the research team

(blind to treatment allocation) for EBT content using the methods and the coding system developed for the initial trial of MATCH (44). We will double-code a subset of the sample to check inter-rater agreement (i.e. κ>0.70).
Efficiency of services. Comparisons will be made between MATCH and UC for the following: Total therapy input by clinicians (in minutes), contact with the CAMHS (in weeks), number of therapy sessions and number of missed therapy sessions.

Secondary outcome measures:

- The Strengths and Difficulties Questionnaire (SDQ) (38) will be collected monthly over the phone. The SDQ for children aged 4 to 16 years is widely used in both research and practice in New Zealand and elsewhere (38). It is was the preferred tool for parents and children in a New Zealand study of child/youth mental health outcome measures (39). It produces a total score with five subscales: emotional symptoms; conduct problems; hyperactivity; peer relationship problems; and prosocial behaviour. Total scores of 17 or above suggest likely cases with mental health disorders (40). It has satisfactory internal consistency, test–retest reliability, and inter-rater agreement (41).
- The Top Problems Assessment (TPA) has been developed to allow the child and family to identify and provide severity ratings for the three top problems of greatest concern to them and track progress in addressing these problems. There is good evidence for reliability, validity, and sensitivity to change (45). It is brief to administer once the top problems are identified (takes <2 minutes to complete on a weekly basis) and can be administered by phone.
- The Development and Well-Being Assessment (DAWBA) (46, 47) will be used to determine the number of diagnoses pre- to post-intervention.
- Child Health Utility (CHU9D) is a measure of health-related quality of life designed specifically for children. There are 9 self-rated items that take fewer than 5 minutes to complete. CHU9D consists of a descriptive system and a set of preference weights, giving utility values for each health state described by the descriptive system. It allows for the calculation of quality adjusted life years (QALYs) for use in cost utility analysis (49).

Other measures
We will collect data on the treatment provided in usual care, the child's/adolescent's psychotropic medication use and clinician rated serious adverse events (SAEs). The Therapist Satisfaction Inventory (TSI) (50) will be used to determine clinicians' satisfaction with therapy. Brief questionnaires will be used to determine treatment satisfaction amongst child/adolescent participants, their parents/caregivers as well as CAMHS managers/team leaders.

Key informant interviews (Amendment Feb 2016):
Key informant interviews will be carried out face-to face (or by phone if face-to-face is not possible) in March and April 2016, and will be overseen by Dr. Sue Crengle, co-principal investigator, with the support of our Māori and Pacific co-investigators.

During the key informant interviews we will explore the following with appropriate study clinicians:

1. Their perspective on the acceptability and appropriateness of MATCH for use with Māori/Pasifika children and young people (CYP).
2. The strengths and limitations of MATCH for use with Māori/Pasifika CYP.
3. What, if any, adaptations clinicians made to the way MATCH was used in the therapy session to ensure that it was delivered in a culturally appropriate way?
4. Any recommendations that could be incorporated for use with Māori/Pasifika CYP if MATCH was implemented nationally.

During the TrACY trial the clinicians providing MATCH have received supervision from experienced MATCH therapists who live and work in the USA. We would also like to explore clinician's experience of this supervision.

5. How useful was the MATCH supervision?
6. Were recommendations made during supervision consistent with the kaupapa (philosophy) and tikanga (practices) used with Māori/Pasifika CYP by the clinician/service?

In-depth individual interviews will be conducted with:

1. Clinicians who work in Kaupapa Māori (KM) and Pasifika (P) services and delivered MATCH in the trial. These clinicians may or may not be of Māori or Pasifika ethnicity themselves. However, because they work in Kaupapa Māori and Pasifika services they are well versed in kaupapa, tikanga and culturally appropriate delivery of care (n = 2 for KM; n = 2 for P).
2. Team leaders of the Kaupapa Māori and Pasifika services (n = 3)
3. Clinicians, working in mainstream services, who delivered MATCH during the trial and have self-identified as Māori or Pasifika ethnicity (n = 1).

With informed consent, all interviews will be digitally (voice) recorded. Transcripts will be made. Participants will be offered the opportunity to review their transcripts. Multiple readings will be undertaken. Thematic analysis, using an inductive approach, will be undertaken to identify common themes and differences across participants. NVivo will be used to manage the qualitative data.

Summary schedule of assessments

Assessment schedule	Baseline	During therapy	Post-intervention	Follow-up	Time	Informant
Demographics	✓				<10 min	Child & parent
SDQ	✓	Monthly	✓	✓	<10 min	Child & parent
BPM	✓	Weekly	✓	✓	<10 min	Child & parent
TPA	✓	Weekly	✓	✓	<2 min	Child & parent
Satisfaction (clients)			✓		<10 min	Child & parent
DAWBA	✓		✓		90 min	Child & parent
CHU9D	✓		✓	✓	<5 min	Child
Medication use	✓		✓	✓	<10 min	Clinician
Treatment description		✓			<15 min	Clinician
SAEs*			✓		<10 min	Clinician
Therapist satisfaction (TSI)			✓		<10 min	Clinician
Delivery of EBT		✓			-	Research team
Efficiency of services			✓		<10 min	Clinician
Satisfaction (manager)			✓		<10 min	Manager

*Some participants will have no SAEs, so this will require no time to complete.

4.13 Research assistants:

We will employ a number of research assistants to conduct many of the assessments. In particular the research assistants will conduct the phone assessments (i.e. SDQ, BPM and TPA) and will assist families to complete the DAWBA. The research assistants will have a health/mental health qualification (ideally NZ registered health professionals) and have experience working with children and adolescents. They will be trained in the administering of the weekly assessments and DAWBA. Ongoing supervision and monitoring will be provided to ensure the fidelity of data collection. Double-data entry will be conducted on a random sample of assessments to ensure accuracy.

4.14 Safety assessments:

Generally, an adverse event (AE) is defined as any unintended, unfavourable clinical sign or symptom, any new illness or disease or a deterioration of existing illness or disease whether or not considered treatment related. For this study clinicians will be asked to record whether a child/adolescent participant experienced any serious adverse events and these will be recorded.

A serious adverse event is one that:

- Results in death;
- Is life-threatening i.e. the patient was, in the opinion of an investigator, at immediate risk of death from the event as it occurred (it does not include an event that, had it occurred in a more severe form, might have caused death);
- Results in persistent or significant disability/incapacity;
- Requires in-patient hospitalisation or prolongs hospitalisation; or,
- Is another medically significant event that, based upon appropriate medical judgement, may jeopardise the patient and may require medical or surgical intervention to prevent one of the outcomes listed above.

All serious adverse events (including increased suicide risk) will be managed by the CAMHS clinicians and their team as per their District Health Board's usual risk management protocols and procedures. All serious adverse events (including increased suicide risk) should be reported to the trial steering committee by the clinician within 24 hours of the clinician being aware of the serious adverse event. This notification should be by phone call and fax to Associate Professor Sally Merry (telephone 09 373 7599 ext 86981 or fax 09 373 7013) and should contain all information available at the time. Further information will be communicated as soon as it becomes available. The trial steering committee will review all serious adverse events as soon as possible. However, the clinical responsibility for the care of the clients will remain with the relevant clinicians at the appropriate CAMHS.

4.15 Statistical considerations:

Study power: Overall, 60 clinicians and 400 patients from a range of CAMHS in New Zealand will provide sufficient power to detect effect sizes of approximately 0.37. This will allow us to detect clinically significant effects sufficient to justify the resources required to introduce MATCH in NZ. We have powered the study so that independent analyses undertaken for Māori and Pacific young people will have sufficient power (80%) to detect moderate effect sizes of between 0.61 to 0.70 as statistically significant (two-tailed α=0.05) (equivalent to the effect sizes found in the USA (32)). This allows for a 15% attrition rate in clinicians and in clients (based on 12.3% attrition rate reported in Weisz et al. (32) and an attrition rate of 10% reported in Merry et al. of a RCT of psychosocial intervention in NZ (51)) and adjustment for the clustering of patients for each clinician (ICC=0.1). We have assumed approximately 8 patients per clinician (approximately 400 patients). If it transpires that fewer clinicians are available within the targeted CAMHS, we will increase the number of participants to ensure that we maintain this level of statistical power. We will recruit approximately 12 clinicians working in Kaupapa Māori services and 12 clinicians working in Pacific services and will investigate effects by service type. The findings will be complemented by data on acceptability, engagement and drop-out rates which will be assessed by ethnicity.

4.15 Amendment (June 2015). Revised sample size and study power: Sample size reduced to at least 200 participants in the overall group, with no specified Māori or Pacific targets.

Because of the difficulties with recruitment, a revised power calculation was carried out in January 2015. By treating the whole group as a single cohort, a sample size of 200 (100 per treatment arm) will provide sufficient power (80%) to detect moderate effect sizes of approximately 0.5 to 0.55 as statistically significant (two-tailed $\alpha=0.05$) for the comparison of outcome measures. These calculations are based on interim analyses of the first 45 completed cases and use a valid estimation of overall variability and acknowledgement of a meaningful change in the primary outcome measure (a different of at least 2 points on externalising and internalising subscales of the BPM).

We were unable to reach our target recruitment in Kaupapa Māori services (11 clinicians, but only 4 who remained in the study and were able to recruit participants) or Pacific services (5 clinicians). Consequently, we are unable to investigate effects by service type.

Analysis of costs: We will compare costs between MATCH and UC using cost-per-hour of clinician time, and will discuss these findings in terms of likely ongoing impact of untreated disorder using information from published studies. Although we are collecting the relevant data, we do not have the resources to undertake a formal analysis of costs in this study. If there are positive results from the study, we will apply for funds to do a detailed cost-benefit analysis.

Analyses:

Intention-to-treat analyses will be carried out and will include all participants who met inclusion criteria and provided and maintained written informed consent prior to treatment commencing.

Per-protocol analyses will include participants who completed the study (i.e. not withdrawn), received therapy as per their allocated treatment group (i.e. not treated "off protocol") and had at least four completed therapy sessions.

We will assess the comparability of baseline of both treatment groups (i.e. MATCH and UC) using descriptive analyses, in terms of age, sex, pre-intervention BPM and SDQ data, diagnosis and ethnicity. Both treatments may vary in content and duration, so that pre-post and follow-up (three months after treatment has ended) analyses are potentially confounded by treatment duration and/or dose. To deal with this, we propose to use the trajectories of change across time on the BPM as our primary outcome measure. Intention to treat analyses will be carried out and will include all participants who meet inclusion criteria, and provide and maintain written informed consent prior to treatment commencing.

Primary outcomes:

i. Clinical outcomes:

Difference between groups on the BPM (internalising and externalising scales) will be compared using differences in trajectories of change across time using a mixed effects regression model with outcome $=a_0$(intercept) + a_1(informant) + a_2(treatment

group) + a_3(time) +a_4(treatment x time) and intercept, informant and time (log day) treated as random effects.

These analyses will be adjusted for:

- Medication use,
- Ethnicity,
- Clinician site, and
- Adjustment for clinician's previous evidence-based therapy training.

The addition of the ethnicity and the treatment x time x ethnicity terms to the model above will allow formal statistical testing of the consistency of the treatment effects across ethnicity groups. It is acknowledged that the power for these comparisons will be much reduced compared to the testing of the overall treatment effect and the possibility of type II error in relation to clinically significant differences is somewhat higher. No comparisons between ethnic groups will be made.

ii. Delivery of EBT. The percentage of EBT content in a random sub-set of sessions assessed by coding audio-taped therapy sessions will be compared between groups using ANOVA following the method used in the USA trial (44).

iii. *Efficiency of services.* Efficiency in delivery of therapy for MATCH and UC will be compared using ANOVA to test for significant differences using the following data which will be collected from the clinical service including:

  - Clinician time (in minutes);
  - Duration of contact with the service (in weeks).
  - Number of therapy sessions;
  - Number of missed therapy sessions.

Secondary outcomes:

- Trajectory of change adjusted as above for the SDQ (total problems score; measured monthly) and Top Problems Assessment (measured weekly);
- Post-treatment diagnoses (number), controlling for pre-treatment diagnoses. A fixed-effects analysis of covariance model will be applied to all children and adolescents for whom we have both pre- and post-treatment data on diagnosis using DAWBA. Where a DAWBA assessment is missing it will be assumed that there has been no change. Remission and reductions of DAWBA diagnoses ("++", "+++" or less), total numbers of DAWBA diagnoses pre- to post-intervention, and diagnoses by treatment arm pre- to post-intervention will also be calculated.
- Changes in the Child Health Utility (CHU9D) assessment will be compared across arms using ANOVA to assess changes between baseline, discharge and at follow-up across treatment groups.

Other measures:

- An evaluation of satisfaction with the treatment (MATCH or UC) compared using ANOVA and rated by:
  a) child/adolescent;
  b) parent/care-giver;
  c) clinicians; and
  d) CAMHS manager/team leaders.

- Clinician rated severe adverse events will be described in each group. The numbers are likely to be too small to allow useful statistical comparisons. Moderate adverse events (gathered by the study research assistants at the three month follow-up) will be described in each group.

- Treatment description (based on the Therapy Procedures Checklist-FR);

- Child's/adolescent's psychotropic medication use;

4.16 Bias:

Due to the nature of the study intervention, this trial can only be participant and assessor-blind. Clinicians will be aware of treatment allocation and some child/adolescent participants may be unblinded to treatment allocation. Research assistants collecting weekly data and will assist families to complete the DAWBA diagnostic assessments will be blind to group allocation.

4.17 Interim analysis:

An interim analysis is planned after child and adolescent participants recruited in the first six months of the study have completed post-intervention assessments. This analysis will compare treatment arms with respect to the primary outcome variable and serious adverse events. The steering committee will consider the relative efficacy and rates of serious adverse events in the two treatment arms and will determine on this basis, if it is appropriate for the study to continue or not.

The DMC will consider the relative safety and efficacy and rates of serious adverse events in the two treatment arms and make recommendations to the Steering Committee regarding continuation or termination of the study.

4.18 Withdrawal criteria:

Participants can withdraw from the study at any time. Those participants that remain in the study, but have dropped out of treatment, will still be followed up wherever possible.

Where a clinician withdraws from the study, a clinician from the same treatment arm will continue to provide the treatment allocated (i.e. either MATCH or usual care). If the participant was randomised to MATCH, and their allocated MATCH clinician withdraws from the study with no one else is available within the team to offer MATCH, then MATCH will be delivered by the MATCH professional support person in that service.

4.19 Ethics committee approval:

Ethics committee approval for this study will be sought from the Health and Disability Ethics Committees and the appropriate locality approval will be obtained from each participating site.

4.20 Dissemination of results:

All study participants (clinicians, children/adolescents and parents/caregivers) who indicated that they would like a brief summary of the results will be sent this summary, along with an outline of their significance and our future research and workforce development plans for MATCH in NZ. Study findings will be discussed at New Zealand meetings, hui and fono as well as at international conferences. Publication of papers in international journals will be sought.

4.21 Trial registration:

The trial has been registered with the Australian New Zealand Clinical Trials Registry ACTRN12614000297628.

5. Administration

5.1 Study management:

The co-principal investigators are primarily responsible for the conduct of the study. The principal investigators will facilitate the trial steering committee and study management committee.

5.2 Trial steering committee:

The trial steering committee will consist of the study investigators and at least two New Zealand experts in the field of child and adolescent mental health. It is responsible for providing strategic guidance for the trial, including developing and maintaining the study design, approval of protocol changes, statistical analyses, presentation and publication of results. The committee will meet at least quarterly (or more frequently if required) and will be conducted using video conference at a time suitable for our American investigators (to allow for those outside of Auckland) to easily access meetings. The committee will review information relating to recruitment and serious adverse events, plus any problems and issues raised by the study management committee. The trial steering committee will review data from the planned interim analysis and recommend whether the study continues or is terminated.

All serious adverse events will be reviewed by at least two or three of the clinicians on the trial steering committee (i.e. Associate Professor Sally Merry, Dr Sue Crengle, Professor John Weisz, Dr Ainsleigh Cribb-Su'a, Dr Jik Loy, Assistant Professor Sarah Kate Bearman, Dr Ana Ugueto, Dr Jenny Herren, Dr Monique Faleafa and Dr Mathijs Lucassen) to establish whether these could be related to the treatment received. If they have any concerns they will forward the information to the full trial steering committee. In addition this information (even if no concerns are raised during the initial review) will be reviewed at the regular trial steering committee meetings.

5.3 Study management committee:

The study management committee consists of Associate Professor Sally Merry, Dr Sue Crengle, Dr Karolina Stasiak, Dr Mathijs Lucassen (1 Oct 2013 - 1 June 2015), and Dr Sarah Hopkins (1 June 2015 – 30 September 2016) who are involved in the daily operation of the study, and will review study materials, deal with study problems, recruitment and logistical issues.

5.4 MATCH training group:

MATCH face-to-face training will be provided by the MATCH trainers from Professor Weisz's team (i.e. Assistant Professor Sarah Kate Bearman, Dr Ana Ugueto and Dr Jenny Herren) in New Zealand. The MATCH trainers will provide weekly group consultation sessions (via telephone or Skype) on the use of MATCH for the duration of study recruitment and the active treatment of child and adolescent participants (estimated to be 15 months). Professor John Weisz will provide oversight to ensure that the MATCH training and consultation provided to New Zealand

clinicians meets the necessary standards. Dr Ainsleigh Cribb-Su'a (or a delegate) will provide support and supervision to the MATCH consultants to help ensure MATCH training and consultation sessions are responsive to the needs to Māori. Dr Monique Faleafa (or a delegate) will provide support and supervision to the MATCH consultants to help ensure MATCH training and consultation sessions are responsive to the needs to Pacific participants in the study.

5.5 Protocol, participant information sheets and other revisions:

Any protocol deviations will be documented. All revisions to the protocol will be discussed and approved by the study management committee. If the revision is an "administrative letter", one of the co-principal investigators will sign it and submit it to the ethics committee for their information. If the revision is an "amendment", one of the co-principal investigators will sign it and submit it to the ethics committee for review and approval prior to implementation. Documentation of approval signed from the chairperson or designee of the ethics committee will be sent to the co-principal investigators.

If an amendment alters the study such that the participant information sheet (PIS) needs to be updated:

- The PIS/consent form will be revised and submitted to the ethics committee for review and approval;
- The revised PIS form will be posted to participants; and,
- The new PIS/consent form will be used for new participants as soon as it is approved by the ethics committee.

5.6 Reporting:

The co-principal investigators will provide annual reports to the ethics committee and the funder (the Health Research Council).

5.7 Record retention and security:

The co-principal investigators will not dispose of any records relevant to this study until 10 years after the study completion. The investigators shall take responsibility for maintaining adequate and accurate hard copy source documents of all data generated during this study and will be securely stored in locked premises at the University of Auckland. Electronic data will be securely stored, using participant codes (except for the form pertaining to demographics) and will be password protected. Participating CAMHS clinicians will have access to clinically relevant data (e.g. a weekly summary of how their participating child/adolescent client is progressing), however they will not have access to the raw data.

5.8 Ownership of data and publication policy:

The data from this study remain the property of the investigators. All publications from this study should be approved by the trial steering committee. It is intended that the results of this study will be presented at appropriate conference/s and published in peer-reviewed journals, regardless of the outcome of the study.

5.9 Confidentiality:

All information generated in this study is considered highly confidential and will not be disclosed to any persons not directly concerned with the study. Documents and data will be stored securely with controlled access to prevent loss, tampering or unauthorised access. Documentation for the study will be managed according to the University of Auckland's research policy. Data will be destroyed after 10 years according to standard research practice.

5.10 Quality control and assurance:

Each study site/CAMHS will be monitored on a regular basis to ensure integrity of the data supplied.

5.11 MATCH training for UC CAMHS clinicians:

Each participating UC CAMHS clinician will be offered the opportunity to receive MATCH training once all participants have been recruited and these participants have finished treatment.

6. References

1. Fergusson DM, Horwood JL. The Christchurch health and development study: review of findings on child and adolescent mental health. Australian and New Zealand Journal of Psychiatry. 2001;35:287-96.
2. Costello JE, Mustillo S, Erkanli A, Keeler G, Angold A. Prevalence and development of psychiatric disorders in childhood and adolescence. Archives of General Psychiatry. 2003;60(8):837-44.
3. Sawyer MG, Arney FM, Baghurst PA, Clark JJ, Graetz RJ, Kosky R, Nurcombe B, Patton GC, Prior MR, Raphael B, Rey JM, Whaites LC, Zubrick SR. The mental health of young people in Australia: key findings from the child and adolescent component of the national survey of mental health and well-being. Australian and New Zealand Journal of Psychiatry. 2001;35.
4. Anderson JC, Williams S, McGee R, Silva PA. DSM-III disorders in preadolescent children: Prevalence in a large sample from the general population. Archives of General Psychiatry. 1987;44(1):69-76.
5. Prime Minister's Chief Science Advisor. Improving the Transition: Reducing Social and Psychological Morbidity During Adolescence. Wellington: Office of the Prime Minister's Science Advisory Committee2011.
6. Belfer ML. Child and adolescent mental disorders: The magnitude of the problem across the globe. Journal of Child Psychology and Psychiatry. 2008;49(3):226-36.
7. Minister of Health. Te Tähuhu - Improving Mental Health 2005-2015: The Second New Zealand Mental Health and Addiction Plan. Wellington: Ministry of Health; 2005.
8. Minister of Health. Te Kökiri: The Mental Health and Addiction Action Plan 2006-2015. Wellington: Ministry of Health; 2006.
9. Ministry of Health. Te Raukura - Mental health and alcohol and other drugs: Improving outcomes for children and youth. Wellington: Ministry of Health; 2007.
10. Weisz JR. Psychotherapy for children and adolescents: Evidence-based treatments and case examples. Cambridge, UK: Cambridge University Press; 2004.
11. Weisz JR, Kazdin AE, editors. Evidence-based psychotherapies for children and adolescents (2nd ed.). New York, NY: Guilford Press; 2010.
12. Weisz JR, Hawley KM, Jensen Doss A. Empirically tested psychotherapies for youth internalizing and externalizing problems and disorders. Child and Adolescent Psychiatric Clinics of North America. 2004;13(2004):729-815.
13. Weisz JR, Jensen Doss A, Hawley KM. Youth psychotherapy outcome research: A review and critique of the evidence base. Annual Review of Psychology. 2005;56(1):337-63.
14. Fixsen DL, Naoom SF, Blase KA, Friedman R, Wallace F. Implementation research: A synthesis of the literature. Tampa, FL: University of South Florida, Louis de la Parte Florida Mental Health Institute, The National Implementation Research Network; 2005.
15. Weisz JR, Jensen-Doss A, Hawley KM. Evidence-based youth psychotherapies versus usual clinical care: A meta-analysis of direct comparisons. American Psychologist. 2006;61(7):671-89.
16. Weisz J, Southam-Gerow M, Gordis E, Connor-Smith J, Chu B, Langer D, McLeod B, Jensen-Doss A, Updegraff A, Weiss B. Cognitive–behavioral therapy versus usual clinical

care for youth depression: an initial test of transportability to community clinics and clinicians. Journal of Consulting and Clinical Psychology. 2009;77(3):383-96.

17. Mental Health Commission. The funding needed for mental health services in New Zealand. Wellington: Mental Health Commission; 1998.

18. The Werry Centre. 2010 Stocktake of Infant, Child and Adolescent Mental Health and Alcohol and Other Drug Services in New Zealand. Auckland: The Werry Centre for Child & Adolescent Mental Health Workforce Development, The University of Auckland; 2011.

19. Peters J. Undergraduate training in child and adolescent mental health: A review. Auckland: Werry Centre for Child and Adolescent Mental Health, University of Auckland; 2003.

20. Strupp HH, Anderson T. On the limitations of therapy manuals. Clinical Psychology: Science and Practice. 1997;4(1):76-82.

21. Westen D, Novotny CM, Thompson-Brenner H. The empirical status of empirically supported psychotherapies: Assumptions, findings, and reporting in controlled clinical trials. Psychological Bulletin. 2004;130(4):631–63.

22. Westen D, Novotny CM, Thompson-Brenner H. The next generation of psychotherapy research: Reply to Ablon and Marci (2004), Goldfried and Eubanks-Carter (2004), and Haaga (2004). Psychological Bulletin. 2004;130(4):677-83.

23. Baxter J, Kingi TK, Tapsell R, Durie M, McGee MA. Prevalence of mental disorders among Maori in Te Rau Hinengaro: the New Zealand Mental Health Survey. Australian & New Zealand Journal of Psychiatry. 2006;40(10):914-23.

24. Adolescent Health Research Group. Te Ara Whakapipi Taitamariki: Maori Specific Findings of Youth2000: The University of Auckland2004.

25. Rasanthan K, Ameratunga S, Chen J, Robinson E, Young W, Wong G, Garret N, Watson PD. A health profile of young Asian New Zealanders who attend secondary school: Findings from Youth2000: The University of Auckland2006.

26. MacMillan HL, MacMillan AB, Offord DR, Dingle JL. Aboriginal health. Canadian Medical Association Journal. 1996;155:1569-78.

27. Trewin D, R M. The Health and Welfare of Australia's Aboriginal and Torres Strait Islander Peoples. Canberra: Australian Bureau of Statistics; 2005.

28. Baxter J, Kokaua J, Wells JE, McGee MA, Browne MAO. Ethnic comparisons of the 12 month prevalence of mental disorders and treatment contact in Te Rau Hinengaro: the New Zealand Mental Health Survey. Australian & New Zealand Journal of Psychiatry. 2006;40(10):905-13.

29. Health Workforce New Zealand 2011 [cited 2011]. Towards the next wave of mental health & addiction services and capability workforce service review report. Available from: http://healthworkforce.govt.nz.

30. Weisz J, Weiss B, Han S, Granger D. Effects of psychotherapy with children and adolescents revisited: a meta-analysis of treatment outcome studies. Psychological Bulletin. 1995;117:450-68.

31. Weisz J, McCarty C, Valeri S. Effects of psychotherapy for depression in children and adolescents: A meta-analysis. Psychological Bulletin. 2006;132(1):132-49.

32. Weisz J, Chorpita B, Palinkas L, Schoenwald S, Miranda J, Bearman S, Daleiden E, Ugueto A, Ho A, Martin J, Gray J, Alleyne A, Langer D, Sotham-Gerow M, Gibbons R, the

Research Network on Youth Mental Health. Testing standard and modular designs for psychotherapy treating depression, anxiety, and conduct problems in youth: A randomized effectiveness trial. Archives of General Psychiatry. 2012;69(3):274-82.
33. Statistics New Zealand. Ethnic population projections: Issues and trends. Wellington: Statistics New Zealand; 2004.
34. Statistics New Zealand. National Ethnic Population Projections: 2006 (base) - 2026 Wellington: Statistics New Zealand; 2010.
35. Ramage C, Bir J, Towns A, Vague R, Cargo T, Niumata-Faleafa M. Stocktake of child and adolescent mental health services in New Zealand. Auckland: The Werry Center for Child and Adolescent Mental Health Workforce Development2005.
36. Fergusson D, Poulton R, Horwood J, Miln B, Swain-Campbell N. Comorbidity and Coincidence in the Christchurch and Dunedin Longitudinal Studies. Wellington: A report for the New Zealand Ministry of Social Development, Ministry of Education and the Treasury; 2003.
37. Donabedian A. The quality of care: How can it be assessed? JAMA. 1988;260(12):1743-8.
38. Goodman R. The Strenghts and Difficulties Questionnaire: A research note. Journal of Child Psychology and Psychiatry. 1997;38:581-6.
39. Merry S, Stasiak K, Parkin A, Seymour F, Lambie I, Crengle S, Pasene-Mizziebo E. Child and youth outcome measures: examining current use and acceptability of measures in mental health services and recommending future directions. Auckland: The Mental Health Research and Development Strategy2004.
40. Goodman R. The Strengths and Difficulties Questionnaire: A research note. Journal of Child Psychology and Psychiatry. 1997;38:581-6.
41. Stone LL, Otten R, Engels RCME, Vermulst AA, Janssens JMAM. Psychometric properties of the parent and teacher versions of the Strengths and Difficulties Questionnaire for 4- to 12-year-olds: A review. Clinical Child & Family Psychology Review. 2010;13:254-74.
42. Chorpita B, Reise S, Weisz J, Grubbs K, Becker K, Krull J, The Research Network on Youth Mental Health. Evaluation of the brief problem checklist: Child and caregiver interviews to measure clinical progress. Journal of Consulting and Clinical Psychology. 2010;78(4):526–36.
43. Achenbach TM. Manual for the Child Behaviour Checklist/4-18 and 1991 profile. Burlington, VT: Department of Psychiatry, University of Vermont1991.
44. Weisz J, Chorpita B, Palinkas L, Schoenwald S, Miranda J, Bearman S, Daleiden E, Ugueto A, Ho A, Martin J, Gray J, Alleyne A, Langer D, Sotham-Gerow M, Gibbons R, the Research Network on Youth Mental Health. Testing standard and modular designs for psychotherapy treating depression, anxiety, and conduct problems in youth: A randomized effectiveness trial - Supplementary Online Content. Archives of General Psychiatry. 2011;Online First:1-4.
45. Weisz J, Chorpita B, Frye A, Ng M, Lau N, Bearman S, Ugueto A, Langer D, Hoagwood K. Youth top problems: Using idiographic, consumer-guided assessment to identify treatment needs and to track change during psychotherapy. Journal of Consulting and Clinical Psychology. 2011;79(3):369-80.
46. Goodman R, Ford T, Richards H, Gatward R, Meltzer H. The development and well-being assessment : Description and initial validation of an integrated assessment of child and

adolescent psychopathology. journal of child psychology and psychiatry. 2001;41(5):645-55.

47. Ford T, Goodman R, Meltzer H. The British child and adolescent mental health survey 1999: The prevalence of DSM-IV disorders. Journal of the American Academy of Child and Adolescent Psychiatry. 2003;42(10):1203-11.

48. Garralda E, Yates P, Higginson I. Child and adolescent mental health service use: HoNOSCA as an outcome measure. The British Journal of Psychiatry. 2000;177:52-8.

49. Stevens KJ. Assessing the performance of a new generic measure of health related quality of life for children and refining it for use in health state valuation. Applied Health Economics and Health Policy. 2011;9(3):157-69.

50. Addis ME, Krasnow AD. A national survey of practicing psychologists' attitudes toward psychotherapy treatment manuals. Journal of Consulting and Clinical Psychology. 2000;68(2):331-9.

51. Merry S, Stasiak K, Frampton C, Shepherd M, Fleming T, Lucassen M. A randomised controlled non-inferiority trial of the effectiveness of SPARX, a computerised self-help intervention for adolescents seeking help for depression. British Medical Journal. 2012;344(e2598).
