# Supplementary material for: Effect of Clinician Training in the Modular Approach to Therapy for Children vs Usual Care on Clinical Outcomes and Use of Empirically Supported Treatments: A Randomized Clinical Trial
Source: JAMA Netw Open. 2020 Aug 17;3(8):e2011799. doi: 10.1001/jamanetworkopen.2020.11799 (PMC7431993; doi:10.1001/jamanetworkopen.2020.11799)
Supplement: Supplement 2. — eTable 1. Individual Diagnoses at Baseline by Treatment Group eTable 2. Clinical Outcomes: Baseline, Post-Intervention and Follow-up Scores—Intention-to-Treat Analyses eTable 3. Changes in Individual Diagnoses by Treatment Group eTable 4. Psychotropic Medication Use by Treatment Arm eTable 5. Adverse Events by Treatment Group eTable 6. Initial Treatment Focus by Treatment Group [file jamanetwopen-3-e2011799-s002.pdf]

Supplementary Online Content

Merry SN, Hopkins S, Lucassen MFG, et al. Effect of clinician training in the Modular Approach to Therapy for Children vs usual care on clinical outcomes and use of empirically supported treatments: a randomized clinical trial. *JAMA Netw Open*. 2020;3(8):e2011799. doi:10.1001/jamanetworkopen.2020.11799

eTable 1. Individual Diagnoses at Baseline by Treatment Group

eTable 2. Clinical Outcomes: Baseline, Post-Intervention and Follow-up Scores—Intention-to-Treat Analyses

eTable 3. Changes in Individual Diagnoses by Treatment Group

eTable 4. Psychotropic Medication Use by Treatment Arm

eTable 5. Adverse Events by Treatment Group

eTable 6. Initial Treatment Focus by Treatment Group

This supplementary material has been provided by the authors to give readers additional information about their work.

© 2020 Merry SN et al. *JAMA Network Open.*

eTable 1. Individual Diagnoses at Baseline by Treatment Group

	MATCH			*Usual Care*		
	**Valid**	***N***	**%**	**Valid**	***N***	**%**
**Depressive disorder**	**58**	**14**	**24.1**	**73**	**13**	**17.8**
**Anxiety disorder / PTSD**	**61**	**30**	**49.2**	**76**	**49**	**64.4**
Separation Anxiety	57	14	24.6	72	23	32.0
Specific Phobia	55	12	21.8	71	24	33.8
Social Phobia	57	8	14.0	69	15	21.7
Panic	54	1	1.9	69	3	4.3
Agoraphobia	54	1	1.9	68	3	4.4
Generalised Anxiety	59	18	30.5	74	21	28.4
PTSD	55	0	0.0	69	2	2.9
OCD	55	2	3.6	70	4	5.7
**Disruptive behaviour disorder**	**51**	**19**	**37.2**	**71**	**19**	**26.7**
Oppositional	50	19	38.0	67	18	26.9
Conduct	51	8	15.7	71	8	11.2
**Other**	**53**	**7**	**13.2**	**74**	**12**	**16.3**
Autistic Spectrum	51	1	2.0	68	0	0.0

© 2020 Merry SN et al. *JAMA Network Open.*

Tics	50	0	0.0	65	2	3.1
Hyperactivity	52	7	13.5	70	9	12.8
Anorexia/Bulimia	51	0	0.0	69	1	1.4

Development and Well-Being Assessment. The presence of disorder was defined as ++ or +++ (50% probability or more). Anxiety/PTSD = Separation Anxiety, Specific Phobia, Social Phobia, Panic, Agoraphobia, Generalised Anxiety, PTSD, OCD. Disruptive behaviour = Oppositional, Conduct.

© 2020 Merry SN et al. *JAMA Network Open.*

eTable 2. Clinical Outcomes: Baseline, Post-Intervention and Follow-up Scores—Intention-to-Treat Analyses

	MATCH			***Usual Care***			***P Value (B – PI)***	***P Value (PI - FU)***
	**Baseline**	**Post-intervention**	**3mo Follow-up**	**Baseline**	**Post-intervention**	**3mo Follow-up**		
**Brief Problem Monitor, N**	96	79	73	103	93	85		
BPM, Total (0 – 26)	12.3 (0.4)	6.3 (0.4)	6.3 (0.4)	11.7 (0.4)	5.8 (0.4)	6.2 (0.3)	0.987	0.776
Internalising (0 – 12)	6.8 (0.2)	3.2 (0.3)	3.2 (0.2)	6.8 (0.2)	3.2 (0.3)	3.3 (0.2)	0.839	0.603
Externalising (0 – 14)	5.5 (0.2)	3.1 (0.3)	3.1 (0.2)	4.9 (0.2)	2.6 (0.2)	2.9 (0.2)	0.805	0.254
**Strengths & Difficulties Questionnaire, N**	97	77	72	103	91	86		
Total difficulties (0 – 40)	19.0 (0.4)	11.5 (0.5)	11.4 (0.4)	18.3 (0.4)	11.5 (0.5)	10.8 (0.3)	0.374	0.509
Internalising (0 – 20)	10.3 (0.3)	6.0 (0.4)	5.7 (0.2)	10.2 (0.3)	5.9 (0.3)	5.5 (0.2)	0.851	0.253
Externalising (0 – 20)	8.7 (0.2)	5.5 (0.3)	5.7 (0.2)	8.1 (0.2)	5.7 (0.3)	5.3 (0.2)	0.199	0.991

All data are reported as means (SE). Abbreviations: BPM, Brief Problem Monitor. P value is for the Treatment*Time interaction.

© 2020 Merry SN et al. *JAMA Network Open.*

eTable 3. Changes in Individual Diagnoses by Treatment Group

	MATCH (n=39)		*Usual Care (n=50)*	
**Change in number of diagnoses**	**n**	**(%)**	**n**	**(%)**
**Gained (+3)**	0	0.0	1	2.0
**Gained (+2)**	1	2.6	3	6.0
**Gained (+1)**	3	7.7	3	6.0
**Same**	15	38.5	11	22.0
**Lost (-1)**	14	35.9	13	26.0
**Lost (-2)**	2	5.1	10	20.0
**Lost (-3)**	3	7.7	6	12.0
**Lost (-4)**	1	2.6	1	2.0
**Lost (-5)**	0	0.0	2	4.0

© 2020 Merry SN et al. *JAMA Network Open.*

eTable 4. Psychotropic Medication Use by Treatment Arm

	MATCH (n=97)		***Usual Care (n=103)***	
	n	**(%)**	***n***	**(%)**
**At Baseline**				
Taking medication	14	(14.4)	9	(8.7)
Missing data	7	(7.2)	5	(4.9)
**During Treatment**				
No change	70	(72.2)	79	(76.7)
Added medication	15	(15.5)	13	(12.6)
Removed medication	1	(1.0)	1	(1.0)
Missing data	11	(11.3)	10	(9.7)

© 2020 Merry SN et al. *JAMA Network Open.*

eTable 5. Adverse Events by Treatment Group

	MATCH (n = 76)	*Usual Care (n=88)*
**Serious Adverse Events**		
Suicide attempt – with or without hospitalisation	4	1
Hospitalisation for non-suicidal self-harm	2	0
**Moderate Adverse Events**		
Hospitalisation for non-mental health issues	4	5
Serious behavioural issues	3	6
Use of respite care	1	2
**TOTAL**	14	14

Examples of hospitalisation responses for non-suicidal self-harm or non-mental health issues: compacted bowel, neck injury & concussion following vehicle accident, overnight stay for blood enzyme deficiency, asthma, playground accident, surgery for kidney issues. Examples of serious behavioural issues: school suspension or expulsion, absconded from home, trouble with police.

© 2020 Merry SN et al. *JAMA Network Open.*

eTable 6. Initial Treatment Focus by Treatment Group

	MATCH (n=97)		*Usual Care (n=103)*		**Total**
	n	**(%)**	***n***	**(%)**	
**Anxiety**	48	(49.5)	67	(65.0)	115
**Depression**	21	(21.6)	11	(10.7)	32
**Conduct**	21	(21.6)	13	(12.6)	34
**Trauma**	1	(1.0)	1	(1.0)	2
**Other [a]**	1	(1.0)	5	(4.9)	6
**Not assessed**	5	(5.2)	6	(5.8)	11

[a] Responses in Other include: "Parent-child relationship", "Eating disorder", "ADHD" (n=2), "Anger management" and "Medical issues, eating fears and Trauma". In secondary post-hoc analyses, we examined the influence of the clinician-reported initial treatment focus on the primary clinical outcome and found no significant influence on the trajectory of change of the parent-rated BPM (p=0.220, for interactions of treatment*time*initial focus – entered as anxiety, depression or conduct only due to the low numbers of trauma participants). In other words, MATCH was comparatively no more or less effective than Usual Care across the primary focus of treatment groups.

© 2020 Merry SN et al. *JAMA Network Open.*
