# Supplementary material for: Effect of Clinician Training in the Modular Approach to Therapy for Children vs Usual Care on Clinical Outcomes and Use of Empirically Supported Treatments: A Randomized Clinical Trial
Source: JAMA Netw Open. 2020 Aug 17;3(8):e2011799. doi: 10.1001/jamanetworkopen.2020.11799 (PMC7431993; doi:10.1001/jamanetworkopen.2020.11799)
Supplement: Supplement 3. — Data Sharing Statement [file jamanetwopen-3-e2011799-s003.pdf]

Data Sharing Statement

Merry. Effect of Clinician Training in the Modular Approach to Therapy for Children vs Usual Care on Clinical Outcomes and Use of Empirically Supported Treatments. *JAMA Netw Open*. Published August 17, 2020.
10.1001/jamanetworkopen.2020.11799

Data
Data available: No

Additional Information
Explanation for why data not available: We did not orgamise to do this when we set up the trial.
